# Inflammation and immune system pathways as biological signatures of adolescent depression—the IDEA-RiSCo study

**DOI:** 10.1038/s41398-024-02959-z

**Published:** 2024-06-01

**Authors:** Valentina Zonca, Moira Marizzoni, Samantha Saleri, Zuzanna Zajkowska, Pedro H. Manfro, Laila Souza, Anna Viduani, Luca Sforzini, Johnna R. Swartz, Helen L. Fisher, Brandon A. Kohrt, Christian Kieling, Marco Andrea Riva, Annamaria Cattaneo, Valeria Mondelli

**Affiliations:** 1 King’s College London, Department of Psychological Medicine, Institute of Psychiatry, Psychology & Neuroscience, London, UK; 2 Department of Pharmacological and Biomolecular Sciences, University of Milan, Milan, Italy; 3 Biological Psychiatry Unit, IRCCS Istituto Centro San Giovanni di Dio Fatebenefratelli, Via Pilastroni, 4, 25125 Brescia, Italy; 4 Department of Psychiatry, Universidade Federal do Rio Grande do Sul, Child & Adolescent Psychiatry Division, Hospital de Clínicas de Porto Alegre, Rua Ramiro Barcelos, 2350—400N, Porto Alegre, RS 90035-903 Brazil; 5 National Institute for Health and Care Research (NIHR) Maudsley Biomedical Research Centre, South London and Maudsley NHS Foundation Trust, King’s College London, London, UK; 6 Department of Human Ecology, University of California, Davis, Davis, CA 95616 USA; 7 King’s College London, Social, Genetic & Developmental Psychiatry Centre, Institute of Psychiatry, Psychology & Neuroscience, London, UK; 8 ESRC Centre for Society and Mental Health, King’s College London, London, UK; 9 Center for Global Mental Health Equity, Department of Psychiatry and Behavioral Health, School of Medicine and Health Sciences, The George Washington University, 2120 L St NW, Ste 600, Washington, DC 20037D USA

**Keywords:** Human behaviour, Psychiatric disorders, Molecular neuroscience, Predictive markers

## Abstract

The biological mechanisms underlying the onset of major depressive disorder (MDD) have predominantly been studied in adult populations from high-income countries, despite the onset of depression typically occurring in adolescence and the majority of the world’s adolescents living in low- and middle-income countries (LMIC). Taking advantage of a unique adolescent sample in an LMIC (Brazil), this study aimed to identify biological pathways characterizing the presence and increased risk of depression in adolescence, and sex-specific differences in such biological signatures. We collected blood samples from a risk-stratified cohort of 150 Brazilian adolescents (aged 14–16 years old) comprising 50 adolescents with MDD, 50 adolescents at high risk of developing MDD but without current MDD, and 50 adolescents at low risk of developing MDD and without MDD (25 females and 25 males in each group). We conducted RNA-Seq and pathway analysis on whole blood. Inflammatory-related biological pathways, such as role of hypercytokinemia/hyperchemokinemia in the pathogenesis of influenza (*z*-score = 3.464, *p* < 0.001), interferon signaling (*z*-score = 2.464, *p* < 0.001), interferon alpha/beta signaling (*z*-score = 3.873, *p* < 0.001), and complement signaling (*z*-score = 2, *p* = 0.002) were upregulated in adolescents with MDD compared with adolescents without MDD independently from their level of risk. The up-regulation of such inflammation-related pathways was observed in females but not in males. Inflammatory-related pathways involved in the production of cytokines and in interferon and complement signaling were identified as key indicators of adolescent depression, and this effect was present only in females.

## Introduction

The increasing prevalence and associated burden of major depressive disorder (MDD) in adolescence has highlighted the urgent need to better understand the biological pathways underlying adolescent depression, with the ultimate goal of developing more effective preventative and treatment strategies [1, 2]. However, the majority of studies on biological mechanisms underlying the onset of depression have focused on adult populations from high-income countries (HICs), whereas far fewer studies have been conducted on adolescent populations and have generally not taken into account the role of environmental risk factors [3–5]. The current study investigates biological signatures by focusing specifically on a sample of adolescents and introducing the novelty of a risk-stratified cohort to disentangle the effects of the presence of MDD from that of environmental risk factors.

Our study is focused specifically on adolescents from a middle-income country (MIC), representing an innovation in the field as the majority of the studies on adolescent depression have been conducted so far in cohorts from HICs, even though 90% of adolescents worldwide live in low- and middle-income countries (LMICs) [6]. Indeed, the different cultural and environmental challenges that might be present in LMICs and MICs (such as low household income, high crime rates, and high school dropout rates) might represent important differences from adolescents in HICs, thus leading also to differences in the biology underlying depression. Filling this gap is of paramount importance to have a global perspective of adolescent depression, as very few studies have focused on LMICs [7, 8]. To this point, it is important to acknowledge the lack of studies in non-European countries also with regards to genome-wide association studies (GWAS), as almost all GWAS have been conducted primarily in populations of European descendants, whereas it is essential that ancestrally diverse populations become better represented [9]. Moreover, polygenic risk scores are based on Eurocentric GWAS, and have been shown to not be equally predictive when applied to non-European populations [10, 11]. Therefore, considering the paucity of studies conducted in underrepresented populations [12–14], further efforts must be made in non-European based populations, and the current project indeed aimed at such a goal.

Most studies on adolescent depression have investigated the risk factors associated with the disorder by focusing on either biological (e.g., cytokines, cortisol, etc.) or environmental (e.g., childhood maltreatment, socio-economic status, etc.) factors. Both play an active role in increasing the risk of developing depression [3, 15–17], and it is critical to understand whether the presence of environmental risk factors independently of MDD is associated with biological abnormalities similar to those present in individuals with MDD. To fill this gap, in the current study, we took advantage of the unique IDEA Risk Stratified Cohort (IDEA-RiSCo) including Brazilian adolescents who were stratified for risk and presence of depression by using a socio-demographic composite risk score previously developed and validated by our team [18]. One of the innovative aspects of the IDEA-RiSCo is that it does not assume that adolescents without depression constitute a homogenous group and therefore uses a risk score (IDEA-RS) to stratify for risk of future depression (low vs. high). The IDEA-RS has been externally validated in both HICs (UK, New Zealand and USA) and LMICs (Nepal, Nigeria and Brazil), and the results of such validation suggested that, although adjustments must be made according to different cultural, economic, and social backgrounds, this prediction model can play a role in the early identification of vulnerable adolescents [19–22]. For the adolescent sample used in this manuscript, no adjustment was needed as the prevalence of individual variables and the network analyses assessing the correlations among these variables revealed a similar pattern when comparing it with the original Brazilian cohort in which the risk score was developed [23]. Moreover, in contrast to previous approaches, where non-cases were identified as those without a psychiatric diagnosis, in this new recruiting strategy, non-cases have been classified as having a high or low risk of developing depression based on empirically-identified environmental risk factors that make them more susceptible to developing MDD in the future.

The identification of biological pathways or markers associated with adolescent depression can take advantage of genome-wide or candidate gene expression studies [24–26]. For instance, genome-wide analysis of RNA-Seq has been widely used as it benefits from being a hypothesis-free approach; thus, no a priori hypothesis must be postulated and a more comprehensive analysis is performed, allowing the identification of novel pathways as well as biomarkers in both clinical and pre-clinical models [27–29]. A large number of studies have used genome-wide analysis as the investigating method for the identification of biological risk factors for the onset of depression, both early and later in life [30–32], and the recent paper by Mariani and colleagues provides a comprehensive overview [33]. Overall, this review showed: a positive correlation between an upregulated expression of pro-inflammatory genes with the presence of MDD together with an altered expression of stress-related genes in MDD patients [33]. However, it is noteworthy to mention that the majority of the studies investigating gene expression in MDD were conducted so far in adult individuals, whereas only a small number of them included adolescents [31, 34–36]. Nevertheless, similar findings between adults’ and adolescents’ transcriptomic profiles have been observed, specifically a dysregulation of inflammatory-related pathways and genes in both adults and adolescents with depression [33]. Among the studies conducted in adolescents, Zhao and colleagues identified enrichments in genes involved in apoptosis, TNF signaling pathway, and NF-kb signaling pathway in MDD adolescents compared with controls [36], and the study by Chiang and colleagues observed an up-regulation of inflammation-related genes and a down-regulation of antiviral-related genes in adolescents with depression compared with their non-depressed peers [34].

The presence of environmental risk factors may also be associated with the presence of biological risk factors, as it has been shown that, for example, increased levels of inflammation are present both in individuals with depression and in those exposed to environmental difficulties, such as childhood trauma [37, 38]. Given these results, in the context of the IDEA-RiSCo project, it is possible to hypothesize that healthy adolescents with increased environmental risk for depression might also have a different biological profile than adolescents without such risk exposure [39].

Given these premises, the main aim of our study was to identify biological pathways associated with the presence of depression in adolescents from Brazil as well as with the risk of developing the disorder based on environmental/sociodemographic risk factors. Furthermore, given the different incidence rate of adolescent depression in males and females, we also aimed to identify possible differences in the biological pathways associated with depression between the sexes. Therefore, we conducted a genome-wide gene expression analysis on whole blood samples from a depression risk-stratified cohort of male and female Brazilian adolescents using the RNA-Seq approach.

## Methods and materials

### Ethical approval

This study was approved by the Brazilian National Ethics in Research Commission (CAAE 50473015.9.0000.5327) and Ethics Committee at King’s College London for secondary data analysis for biological measures (LRS-17/18-8327). Informed consent was obtained from adolescents and their caregivers prior to taking part in the study. This study is part of the wider IDEA-RiSCo study details of which can be found in our previous publications [23, 40, 41]. For the purpose of this article, we only report on methods relevant to this study.

### Sample recruitment

The sample recruitment process have been described in our previous paper [23] and also in the Supplementary Methods. Briefly, we recruited 150 adolescents, aged 14–16 years, from public schools in Porto Alegre, Brazil, stratified for risk of depression using the IDEA risk score (IDEA-RS) [18]. The IDEA-RS consists of 11 sociodemographic variables obtained directly from the adolescent: biological sex, skin color, drug use, school failure, social isolation, fight involvement, relationship with mother, relationship with father, relationship between parents, childhood maltreatment, and ran away from home. The use of psychotropic medication within the last 30 days was an exclusion criterion. Questions about any lifetime use of alcohol, tobacco, cannabis, cocaine, and inhalants were combined into one variable using the OR rule, generating a binary variable for analyses and this was included and represented by the term “drug use” for the IDEA-RS. The participants were divided into three groups: (1) non-depressed low-risk (LR) adolescents (*n* = 50), who were those scoring equal to or below the 20th percentile of the IDEA-RS; (2) non-depressed high-risk (HR) adolescents (*n* = 50), who were those scoring equal to or above the 90th percentile of the IDEA-RS and who did not meet the diagnostic criteria for MDD; (3) depressed high-risk (MDD) adolescents (*n* = 50), who were those scoring equal to or above the 90th percentile of the IDEA-RS and who met the diagnostic criteria for MDD. To optimize the recruitment process and increase the probability that diagnostic criteria for depression were met in the MDD group, but not in the LR and HR groups, during the school screening adolescents also completed the Patient Health Questionnaire—adolescent version (PHQ-A). Adolescents with a PHQ-A ≤ 6 were considered for further assessment for the LR/HR groups, and those with a PHQ-A ≥ 10 for the MDD group [23]. The clinical assessment of the participants was performed using the Kiddie Schedule for Affective Disorders and Schizophrenia (K-SADS). Each of these groups included 25 females and 25 males, with a total of 75 females and 75 males.

### Biological sample collection and RNA-Seq

Peripheral venous blood samples were collected at baseline on the same day as the clinical assessment in 2.5 ml PAXgene Blood RNA tubes (PreAnalitix, Qiagen/BD Company). Nucleic acid purification from PAXgene tubes was performed by using the PAXgene Blood miRNA kit (Qiagen, Hilden, Germany; Cat No./ID: 763134). The quality of the RNA was assessed by Agilent 2100 Bioanalyzer (Agilent, Santa Clara, United States) with RNA 6000 Nano Kit to measure the RNA integrity number (RIN). RNA-Seq was performed starting from 150 ng and by using the Illumina Stranded mRNA Prep Ligation Kit following the manufacturer’s instruction. The libraries were sequenced on the NextSeq 550 Illumina platform using High Output Kit v2.5 (150 Cycles) paired-ended, read length 74.

### Biostatistical analysis

Raw read counts were quantified at the transcript level by using Salmon (v 1.4.0) in the quasi-mapping mode, consisting of two steps: (1) indexing of the transcriptome and (2) quantification of the set of raw sequencing reads (FASTQ format) [42]. The first step was building a decoy-aware transcriptome where the entire human genome was concatenated to the end of the human transcriptome and subsequently indexed with the chromosome names following the instructions reported in https://combine-lab.github.io/alevin-tutorial/2019/selective-alignment/. Both genome and transcriptome (Release 38 (GRCh38.p13)) were downloaded from https://www.gencodegenes.org/human/. The second step consisted of the quantification of the paired-end reads directly against the index built during the previous step.

Transcript-level differential expression was assessed using the negative binomial model (Wald test) implemented in DESeq2 (v1.30.1) in R to compare the three risk groups divided or not by sex, and accounting for RNA-Seq run as a covariate. In the comparisons between the groups (for example MDD vs. HR) that were performed, the third group (LR in the example) was excluded from the analysis. Before differential expression analysis, the transcript set was filtered for library size, and transcripts with less than 10 counts across all samples were excluded. Differentially expressed transcripts were identified by applying a fold change (FC) cut-off of ± |1.2| and an unadjusted *p* value < 0.05.

Transcripts differentially modulated across groups were then imported into Ingenuity Pathway Analyses Software (IPA) to identify associated biological pathways (*p* < 0.05). The IPA software does not apply a Bonferroni correction or any other correction because of a possible overcorrection of the results, leading to a high false negative rate accordingly to the IPA user manual. Moreover, it is recommended to not use multiple testing corrections when analyzing canonical pathways, and this advice was followed in the current paper. Lastly, the IPA software allowed us to select for the cell type to be considered for the analysis; therefore, blood cells were selected as possible and unique sources of results, thus allowing us to take into account that a bulk RNA-seq had been performed. The reference gene set used was the same used for the DE transcripts analysis. Further information regarding IPA software is reported in the Supplementary Methods.

The pathways reported in the figures are only those presenting a *z*-score >2 and <−2, whereas all the other pathways are reported in the Supplementary Tables. This was not used as a cut-off but as a selection criterion for the graphical representation of the pathway analysis.

For the RNA Sequencing power analysis, by using the ssizeRNA package, a sample size of 50 per group was shown to have a power of >90% to detect significant differences in the expression of 100 transcripts, with FC of 2, FDR = 0.05, and considering 15,000 as the number of detected transcripts and 0.1 as the dispersion parameter for each gene [43]. On the other hand, no power calculation has been performed for the pathway analysis.

## Results

### Sociodemographic characteristics of the IDEA-RiSCo

The sociodemographic characteristics of the IDEA-RiSCo study have been previously described elsewhere [23]. The basic sociodemographic and clinical characteristics of the adolescents are reported in Tables 1 and S1 when divided by sex.

**Table 1:** Sociodemographic characteristics of the IDEA-RiSCo.

	**Low risk Mean (SD)**	**High risk Mean (SD)**	**MDD Mean (SD)**
Age (years)	15.4 (0.8)	15.8 (0.8)	15.8 (0.7)
Body Mass Index	22.1 (5.5)	22.4 (4.8)	22.7 (3.9)
IDEA-RS (%)	1.33 (0.3)	8.21 (1.6)	9.24 (5.6)
PHQ-A	2.82 (1.5)	3.96 (1.6)	18.82 (4.5)
CTQ	29.16 (3.3)	38.08 (8.2)	51.56 (13.2)
	**Low risk** ***n*** **(%)**	**High risk** ***n*** **(%)**	**MDD** ***n*** **(%)**
Sex, female	25 (50.00)	25 (50.00)	25 (50.00)
Skin color, non-white	22 (44.00)	26 (52.00)	26 (52.00)
Meets friends	49 (98.00)	40 (80.00)	30 (60.00)
School failure	0 (0.00)	29 (58.00)	25 (50.00)
Ran away	1 (2.00)	3 (6.00)	13 (26.00)
Any drug use	29 (58.00)	44 (88.00)	47 (94.00)
Fights	0 (0.00)	20 (40.00)	27 (54.00)
Relationship with father (mean, SD)	4.52 (0.79)	2.48 (1.22)	2.00 (1.18)
Relationship with mother (mean, SD)	4.78 (0.54)	3.92 (1.01)	3.14 (1.14)
Relationship between parents (mean, SD)	4.18 (1.08)	2.38 (1.23)	1.94 (1.04)
Childhood maltreatment—None	50 (100.00)	1 (2.00)	0 (0.00)
Childhood maltreatment—Probable	0 (0.00)	12 (24.00)	4 (8.00)
Childhood maltreatment—Severe	0 (0.00)	37 (74.00)	46 (92.00)

*SD* standard deviation, *IDEA-RS* IDEA Risk Score, *PHQ-A* Patient Health Questionnaire for Adolescents, *CTQ* Childhood Trauma Questionnaire.

### Transcripts and pathways differentially expressed in adolescents with MDD

To identify biological signatures differentially associated with the presence or the risk of developing depression, we first investigated the transcripts differentially expressed in adolescents with MDD compared with the two non-depressed groups separately (HR and LR). The numerosity of the transcript, before and after applying the filters as described in the “Methods”, is reported in Table S2.

The lists of differentially expressed (DE) transcripts are reported in the Supplementary Materials. We found 313 DE transcripts when comparing MDD adolescents with the HR group (173 up-regulated and 140 down-regulated) and 461 DE transcripts when comparing MDD with the LR group (206 up-regulated and 255 down-regulated). Starting from these DE transcripts, we performed the pathway analysis; the pathways reported in the figures are only those presenting a *z*-score ≥2 and ≤−2, whereas all the other pathways are reported in the Supplementary Tables.

**Fig. 1:**
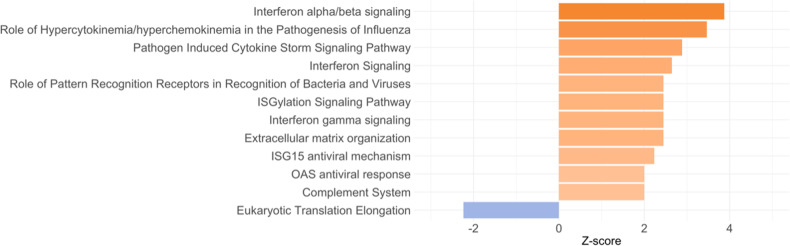
Pathways differently modulated in adolescents with current major depressive disorder (MDD) compared with adolescents at high risk (HR) for future depression (both males and females). For graphical purposes, only pathways with *z*-scores ≥2 or ≤−2 are plotted on this figure; pathways colored in orange represent a *z*-score ≥2, which means a predicted activation of that pathway in MDD adolescents compared with HR adolescents. On the other hand, pathways colored in blue represent z-scores ≤−2, which means a predicted inactivation of that pathway in MDD adolescents compared with HR adolescents. Pathways without the *z*-score have not been reported in the figure but are reported in Supplementary Table S3.

**Fig. 2:**
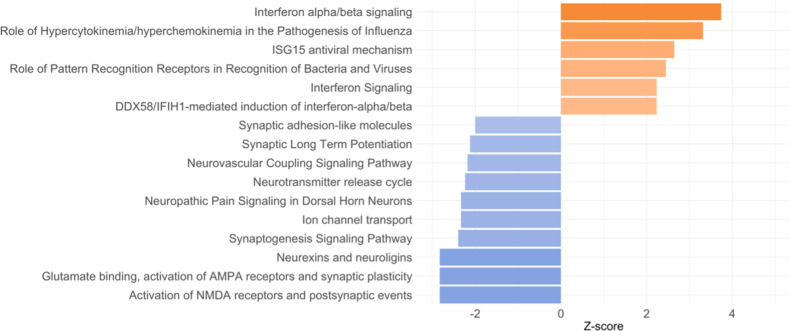
Pathways differentially modulated in adolescents with current major depressive disorder (MDD) compared with adolescents at low risk (LR) for future depression (both males and females). For graphical purposes, only pathways with *z-score ≥2 or ≤−2* are plotted on this figure; pathways colored in orange represent *z*-scores ≥2, which means a predicted activation of that pathway in MDD adolescents compared with LR adolescents. On the other hand, pathways colored in blue represent *z*-scores ≤−2, which means a predicted inactivation of that pathway in MDD adolescents compared with LR adolescents. Pathways without a *z*-score have not been reported in the figure but are reported in Supplementary Table S4.

When comparing MDD with HR adolescents, 40 pathways were found to be significantly modulated (*p* < 0.05) (Fig. 1 and Table S3). Specifically, the most significant were *Interferon alpha/beta signaling* (*z*-score = 3.873, *p* < 0.001), *role of hypercytokinemia/hyperchemokinemia in the pathogenesis of influenza* (*z*-score = 3.464, *p* < 0.001), *interferon signaling* (*z*-score = 2.646, *p* < 0.001), *role of pattern recognition receptors in recognition of bacteria and viruses* (*z*-score = 2.449, *p* < 0.001), and *complement system* (*z*-score = 2, *p* = 0.002). When comparing the MDD with the LR group, we identified 80 pathways that were significantly modulated, and among them, we observed an up-regulation of *interferon alpha/beta signaling* (*z*-score = 3.742, *p* < 0.001), *role of hypercytokinemia/hyperchemokinemia in the pathogenesis of influenza* (*z*-score = 3.317, *p* < 0.001), *interferon signaling* (*z*-score = 2.236, *p* = 0.002), and *role of pattern recognition receptors in recognition of bacteria and viruses* (*z*-score = 2.449, *p* = 0.002) (Fig. 2 and Table S4). Moreover, we identified some biological pathways that were down-regulated in MDD adolescents compared with the LR group, such as *calcium signaling* (*z*-score = −1.508, *p* < 0.001) and *glutamate receptor signaling* (*z*-score = −1.633, *p* < 0.001).

An exploratory analysis to identify the overlap between the DE transcripts of MDD vs. HR and MDD vs. LR was performed, showing 107 DE transcripts in common, and Pearson’s correlation analysis was done by considering positive and negative FCs together, only positive FCs and only negative FCs (Supplementary Fig. 1). For all three analyses, FCs from both the comparisons were highly correlated (rho > 0.83, *p* < 0.001).

**Fig. 3:**
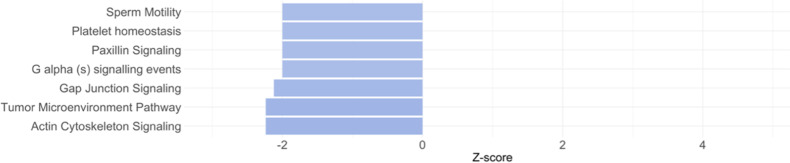
Pathways differently modulated in adolescents at high risk (HR) for future depression compared with adolescents in the low risk (LR) group (both males and females). For graphical purposes, only pathways with *z*-scores ≥2 or ≤−2 are plotted on this figure; pathways colored in blue represent *z*-scores ≤−2, which means a predicted inactivation of that pathway in HR adolescents compared with LR adolescents. Pathways without a *z*-score have not been reported in the figure but are reported in Supplementary Table S5.

We also investigated whether there were differences in terms of pathways differentially modulated comparing HR and LR individuals. After the identification of the DE transcripts (192, 63 up-regulated and 129 down-regulated), we identified 48 pathways that were significantly modulated (Table S5). The pathways most significantly modulated in this comparison were all inactivated in the HR adolescents, and their biological profile was heterogenous as they included signaling of different mechanisms associated for example with cell motility (e.g., *paxillin signaling*, *actin cytoskeleton signalin*g, *integrin signaling*—Fig. 3). The heatmap of the activation Z score of statistically significantly enriched pathways for the comparison between MDD vs. HR, MDD vs. LR and HR vs. LR is reported in the Supplementary Fig. 2.

### The role of biological sex in the differential expression profile and pathways in depressed adolescents

**Fig. 4:**
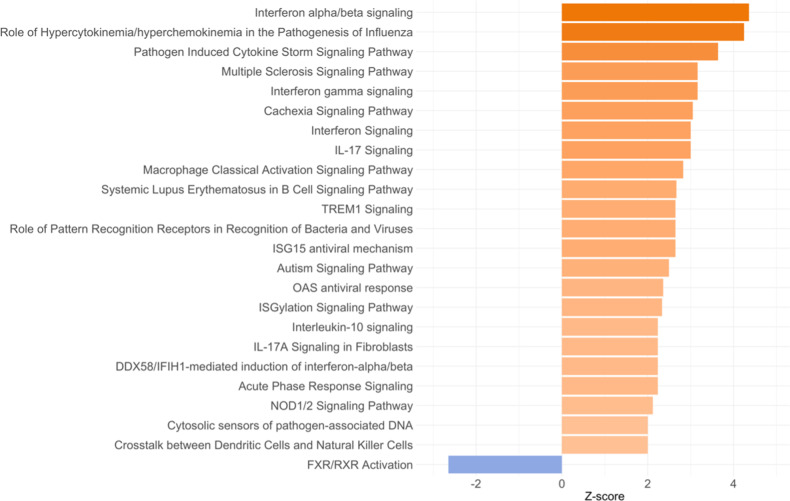
Pathways differently modulated in female adolescents with current major depressive disorder (MDD) compared with the high risk (HR) female group. For graphical purposes, only pathways with *z*-scores ≥2 or ≤−2 are plotted on this figure; pathways colored in orange represent *z*-scores ≥2, which means a predicted activation of that pathway in MDD adolescents compared with HR adolescents. On the other hand, pathways colored in blue represent z-scores ≤−2, which means a predicted inactivation of that pathway in MDD adolescents compared with HR adolescents. Pathways without a *z*-score have not been reported in the figure but are reported in Supplementary Table S6.

To investigate the differences in the transcriptomic profile associated with biological sex, we analyzed the transcriptomic profile in males and females separately, by comparing the transcriptome of MDD adolescents versus both the two non-depressed groups (HR and LR). The transcripts differentially expressed for each of the comparisons in females presented a slightly higher numerosity compared with males (399 vs. 310 for the MDD vs. HR comparison, and 471 vs. 377 in the MDD vs. LR in females and males, respectively). In the comparison of MDD females with those in the HR group, 73 pathways were identified as significantly modulated (Fig. 4 and Table S6), and among them, we observed a modulation towards the activation of *Interferon alpha/beta signaling* (*z*-score = 4.359, *p* < 0.001), *role of hypercytokinemia/hyperchemokinemia in the pathogenesis of influenza* (*z*-score = 4.243, *p* < 0.001), *interferon signaling* (*z*-score = 3, *p* < 0.001), *role of pattern recognition receptors in recognition of bacteria and viruses* (*z*-score = 2.646, *p* < 0.001), *IL-17 signaling* (*z*-score = 3, *p* = 0.004), and *acute phase response signaling* (*z*-score = 2.236, *p* = 0.030). In the comparison of MDD vs. LR in the female group, we observed 46 pathways that were significantly modulated, and among them, we observed *phagosome formation* (*z*-score = −0.18, *p* < 0.001), *STAT3 pathway* (*z*-score = −0.707, *p* = 0.001), *CDK5 signaling* (*z*-score = −1.134, *p* = 0.030), and *natural killer cell signaling* (*z*-score = 1, *p* = 0.035) (Table S7). On the other hand, fewer pathways were observed to be modulated in MDD males compared with males in both risk groups; these are reported in Tables S9 and S10.

We also investigated whether there were differences in terms of pathways differentially modulated between HR and LR adolescents in males and females separately. In males (359 DE transcripts, 215 up-regulated and 144 down-regulated), 26 pathways were significantly modulated (Table S11), whereas in females (504 DE transcripts, 197 up-regulated and 307 down-regulated), 60 pathways were found to be significantly modulated, such as the *STAT3 Pathway* (*z*-score = 1.134, *p* < 0.001), *Inflammasome pathways* (*z*-score = −2.236, *p* < 0.001), *Complement cascade* (*z*-score = −1.143, *p* < 0.001), and *hepatocyte growth factor (HGF) signaling* (*z*-score = 2, *p* = 0.042) (Table S8).

## Discussion

Our study showed the activation of inflammation and immune system-related pathways, such as role of hypercytokinemia and hyperchemokinemia in the pathogenesis of influenza, interferon and interferon alpha/beta signaling, and complement system in adolescents with depression when compared with adolescents who do not meet current clinical criteria for depression, regardless of whether they are high- or low-risk for future development of MDD. Moreover, such activation of pro-inflammatory pathways was observed only in females with MDD, whereas no inflammation-related pathways were observed in males.

When considering each risk group comprising both males and females, we observed an enrichment in pathways associated with inflammation and immune system activation in adolescents with depression compared with their non-depressed peers classified for different risk degrees of developing the disorder. The link between depression and inflammation has been widely studied in the recent literature, both in adulthood and adolescence [24, 37, 38, 44, 45], and our data are consistent with these reports as they showed an up-regulation of the interferon (IFN) and interferon alpha/beta signaling and of the complement signaling pathway. Indeed, previous literature has observed a significant association between depression and interferon α/β signaling pathways’ genes in large population-based samples [46, 47]. The activation of the interferon pathway in depression is also consistent with studies reporting an increased prevalence of clinically relevant depression in patients receiving IFN-I therapies (IFN-α for hepatitis C or IFN-β for multiple sclerosis) [29, 48]. The complement system pathway was also shown to be up-regulated in adolescents with depression compared with the HR group. Given its role in activating the inflammatory response and considering the link between inflammation and depression, it is possible to suggest that the activation of the complement system in adolescents with depression might be part of the general inflammatory response shown in depression. Interestingly, a previous study, considering both a cohort of adult men from HICs and an animal model of depression, has shown that the complement system can influence IFN signaling [49]. In particular, the complement component 3 has been shown to mediate systemic IFN-β-induced changes in neuroinflammation and behavior of mice subjected to unpredictable chronic mild stress and a similar correlation has also been shown in humans, as increased expression levels of IFN-I stimulated genes and significant correlations with complement 3 and inflammatory markers have been observed in the prefrontal cortex of individuals with depression who died by suicide [49].

We also observed an up-regulation of the hypercytokinemia/hyperchemokinemia pathway in adolescents with MDD, which is in line with the literature since increased levels of pro-inflammatory cytokines have been observed in adolescents with depression [3, 15, 50]. Indeed, recent meta-analyses have reported higher levels of TNF-α, CRP, and IL-6 levels in adolescents with depressive disorders when compared with control individuals [3, 51]. Therefore, our results showing a modulation towards the activation of pro-inflammatory pathways in adolescents with depression compared with HR adolescents are in line with the previous literature [52–54]. Moreover, these results observed in adolescents are also consistent with the literature showing an overall activation of inflammation in adult individuals with depression compared with controls [37, 38, 55, 56].

It is noteworthy to mention that the activation of the pro-inflammatory pathways of IFN and interferon alpha/beta signaling as well as hypercytokinemia/hyperchemokinemia pathway was observed also when analyzing female adolescents only, whereas no such pro-inflammatory signatures were observed in males only. Thus, these results suggest that inflammation might be involved in the onset of depression in adolescent females only, and this is in line with previous studies showing increased levels of inflammation and pro-inflammatory cytokines in females with depression compared with control female individuals [57, 58]. As there were no differences in BMI and the sociodemographic variables between males and females, the results we observed in males are not accounted for such variables.

The activation of inflammation was slightly less evident when comparing the MDD and LR groups, suggesting that the risk stratification applied in this study might play a role in differentiating the transcriptomic profile of those adolescents and may partly explain inconsistent findings from previous studies. Furthermore, our results suggest that other pathways different from inflammation, such as calcium signaling and glutamate receptor signaling, which were shown to be down-regulated in the MDD vs. LR comparison, might play a role in distinguishing adolescents with MDD from healthy adolescents without specific sociodemographic risk factors. Moreover, it is important to highlight that the individuals in the MDD and HR groups all scored higher than the 90th percentile in the IDEA-RS and the IDEA-RS were similar between HR and MDD, with no significant difference between their scores (8.21 vs. 9.24, *p* > 0.05, see Table 1). Therefore, the differences between MDD and HR adolescents do not appear to be due to differences in the risk score but rather to the absence or presence of depression.

On the other hand, the differences in the transcriptomic profiles of the HR and LR groups were mainly related to pathways belonging to very diverse biological mechanisms and thus a specific biological signature was not identified as able to distinguish HR and LR adolescents, specifically as compared to what we observed for the comparisons with the MDD group, where inflammatory-related signatures were driving the results. Indeed, although increased cytokine levels and increased inflammation have also been previously related to a higher risk of depression in adolescents [50, 59], we did not find specific up-regulation of immune pathways or other specific biological pathways in the HR vs. LR adolescents. This might suggest that the biological differences between non-depressed adolescents stratified by risk could be less evident and detectable, also given the fact that the risk score took into account several socio-demographic variables and this heterogeneity might be mirrored also by a biological heterogeneity [18]. Therefore, it is possible that specific environmental risk factors may be more relevant than others in modulating biological pathways involving the immune system; consistent with this hypothesis, increased inflammation has been associated with an increased risk of MDD in particular when associated with early-in-life trauma rather than other environmental risk factors [39].

However, it is noteworthy to highlight that this cohort was represented by adolescents from a MIC, thus representing an underrepresented group in literature, as the majority of studies conducted so far have focused their attention on adolescents from HICs. Among the studies conducted in LMICs, Perez-Sanchez investigated depression in a cohort of 14–19-year-olds in Mexico City and observed increased levels of pro-inflammatory cytokines in depressed individuals compared with controls [60]. In contrast, no increased inflammation was shown in two different studies investigating adolescent depression in the Philippines and Chile [7, 8]. An interesting explanation of such results, which are opposite to most of the other studies conducted in HICs, was that chronic inflammation levels might be lower in environments characterized by higher prevalence of infectious diseases, such as the Philippines [7]. Indeed, using risk factors and evidence from HICs and applying this to LMICs is problematic for three main reasons: (1) risk factors may be interpreted differently across different cultural contexts; (2) risk factors may have different impacts across different populations; and (3) the feasibility of assessing and measuring specific risk factors varies across different settings [61]. Thus, possible discrepancies in our results compared to the literature might be due to the different cultural and socioeconomic background of our sample, although the results on inflammation indeed strengthen the role of such biological systems as underlying adolescent depression.

This study has some limitations. Firstly, this is a cross-sectional study, thus it is not possible to ascertain whether the biological signatures identified in the risk groups will predict the development of depression later during adolescence. This might also explain the results observed in the HR vs. LR comparison. However, this study has the advantage of leveraging a unique risk-stratified cohort of adolescents and of addressing the issues of comparing adolescents with depression with a potentially widely heterogeneous sample of controls. Indeed, the recruitment of this cohort was based on a careful phenotypic characterization of the three groups with marked differences in terms of exposure to risk factors and manifestation of symptomatology. A second limitation is that no adjustments have been made to correct RNAseq analysis for cell-type heterogeneity, as statistical deconvolution methods estimating the proportions between cell types and infer cell type-specific expression profiles commonly use as the input a reference molecular profile of the individual blood cell types and, to the best of our knowledge, no reliable datasets are available to be used as a reference. A third limitation is that an insufficient number of transcripts survived the FDR correction and thus were not suitable for the subsequent pathway analysis. Therefore, for the pathway analysis we used DE transcripts with an unadjusted *p* value. However, it is important to clarify that it is not unusual for such a small number of DE transcripts to survive the FDR correction in genome-wide gene expression analysis, specifically when genome-wide gene expression analyses are performed in clinical cohorts [62, 63]. Then, as few or no transcripts survived the FDR correction, it is possible to expect some false positive DE transcripts. Moreover, a further limitation might be represented by the fact that no FDR correction has been applied to IPA *p* values following the instruction of the IPA users’ manual. It is also important to acknowledge that the three groups have not been analyzed in a single model, but a comparison between two groups (for example MDD and HR) have been performed excluding one of the three groups (i.e., the LR group in this example comparison). Moreover, the small sample size represented a limitation. However, the small sample size has been counterbalanced by focusing on more homogeneous groups and employing comprehensive clinical assessment procedures, which is not always the case in large samples that frequently rely only on short, self-reported measures. Furthermore, as explained in one of our previous papers [23], the requirement of a high IDEA-RS for the MDD group, included in our design to allow for direct comparisons with the HR group, inevitably makes the former less representative of the overall population of youths with depression.

Lastly, the differences observed in the gene expression of the three groups might also be associated with the different rates of “any drug use” in the screening phase, as LR participants had lower lifetime drug use in comparison to HR and MDD participants (who did not differ from each other). However, it is also important to highlight that in the clinical phase we evaluated participants with the K-SADS-PL module on alcohol and other substance use disorders and participants were excluded if they met lifetime diagnosis for substance use disorders.

In conclusion, inflammation and immune system-related pathways were identified as leading biological pathways associated with the presence of adolescent depression in a risk-stratified cohort of 14–16-year-old adolescents in Brazil. This modulation toward their up-regulation was observed in adolescents with depression compared with adolescents without MDD both at high and low risk of developing the disorder, although such activation was more evident when comparing adolescents with depression with the HR group. This result might represent an important observation as both depressed and HR adolescents were classified at high risk of developing MDD according to the IDEA-RS, suggesting also that the HR adolescents, even though they have been exposed to risk factors, might have resilience/compensatory mechanisms which may protect them from developing depression. Therefore, enhanced activity of inflammation might represent one of the biological pathways characterizing the presence of depression in adolescents from Brazil. Moreover, our data supported the role of immune-related pathways in the pathophysiology of adolescent depression particularly in girls, suggesting a possible explanation for the increased incidence of depression in adolescent females compared with males. Future longitudinal studies will be required to understand whether the absence of inflammation represent a resilience mechanism for adolescents at high risk who may not develop depression later on.

### Supplementary information

Supplementary Figure 1

Supplementary Figure 2

Supplementary Tables

Supplementary Methods

Supplementary Results

## Data Availability

Data will be made available upon request.
