# Supplementary material for: Inflammation and immune system pathways as biological signatures of adolescent depression—the IDEA-RiSCo study
Source: Transl Psychiatry. 2024 Jun 1;14:230. doi: 10.1038/s41398-024-02959-z (PMC11144232; doi:10.1038/s41398-024-02959-z)

*Supplementary figure 1. Scatterplot for fold changes of DEGs common in both MDD vs HR and MDD vs LR comparisons. One transcript was excluded from the analyses because of the very high FC (FC of TBC1D3 in MDD vs HR= 8.96, in MDD vs LR= 13.97) that would have biased the correlation analyses. Pearson’s correlation analysis was done by considering positive and negative FCs together, only positive FCs and only negative FCs. The dotted line represents the diagonal, each dot a transcript. If a point lies on the diagonal, it means the FC of MDD vs HR and MDD vs LR is equal, if it is below the diagonal the FC is greater for MDD vs HR, if it is above the diagonal the HR is greater for MDD vs LR.*

*For all three analyses, FCs from both the comparisons were well correlated (rho> 0.83, P-val< 0.001). Considering all DEGs and those with positive FCs, DEGs were equally distributed both above and below the diagonal while for negative FCs they were mainly below the diagonal.*


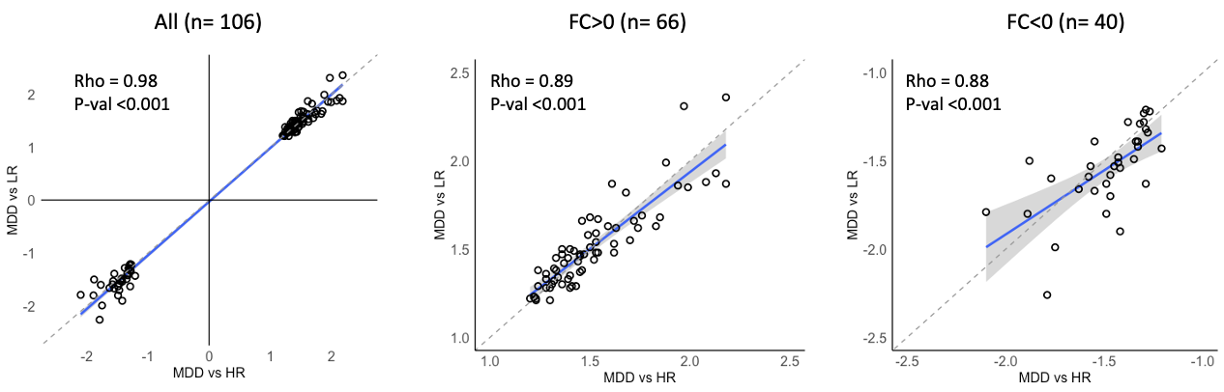

Supplement: Supplementary file 1 — Supplementary Figure 1 [file 41398_2024_2959_MOESM1_ESM.docx]
