# Supplementary figures and images for: Inflammation and immune system pathways as biological signatures of adolescent depression—the IDEA-RiSCo study
Source: Transl Psychiatry. 2024 Jun 1;14:230. doi: 10.1038/s41398-024-02959-z (PMC11144232; doi:10.1038/s41398-024-02959-z)

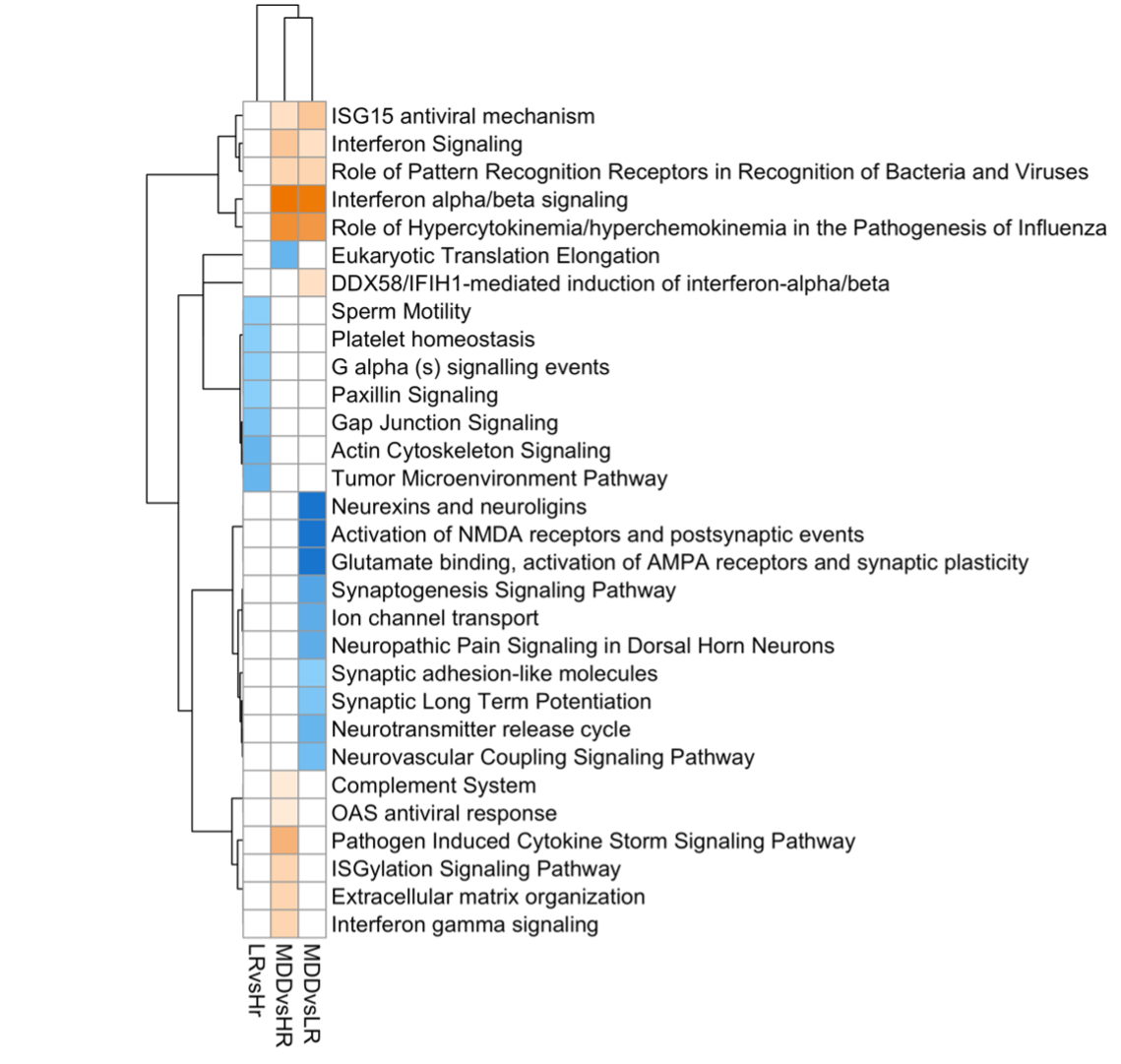

Supplement: Supplementary file 2 — Supplementary Figure 2 [file 41398_2024_2959_MOESM2_ESM.png]
