# Supplementary material for: Inflammation and immune system pathways as biological signatures of adolescent depression—the IDEA-RiSCo study
Source: Transl Psychiatry. 2024 Jun 1;14:230. doi: 10.1038/s41398-024-02959-z (PMC11144232; doi:10.1038/s41398-024-02959-z)
Supplement: Supplementary file 3 — Supplementary Tables [file 41398_2024_2959_MOESM3_ESM.docx]

# SUPPLEMENTARY TABLES

[SUPPLEMENTARY TABLES 1](#_Toc161393402)

[Table S1. Sociodemographic characteristics of the IDEA-RiSCo divided by sex. 2](#_Toc161393403)

[Table S2. Numerosity of the transcripts, before and after applying the filters. 4](#_Toc161393404)

[Table S3. 40 pathways MDD vs HR (p-value < 0.05). 5](#_Toc161393405)

[Table S4. 80 pathways MDD vs LR (p-value < 0.05) 7](#_Toc161393406)

[Table S5. 48 pathways HR vs LR (p-value < 0.05) 11](#_Toc161393407)

[Table S6. 73 pathways females MDD vs females HR (p-value < 0.05) 13](#_Toc161393408)

[Table S7. 46 pathways females MDD vs females LR (p-value < 0.05) 17](#_Toc161393409)

[Table S8. 60 pathways females HR vs females LR (p-value < 0.05) 20](#_Toc161393410)

[Table S9. 26 pathways males MDD vs males HR (p-value < 0.05) 23](#_Toc161393411)

[Table S10. 50 pathways males MDD vs males LR (p-value < 0.05) 24](#_Toc161393412)

[Table S11. 26 pathways males HR vs males LR (p-value < 0.05) 27](#_Toc161393413)

## Table S1. Sociodemographic characteristics of the IDEA-RiSCo divided by sex.

SD: Standard Deviation; IDEA-RS: IDEA Risk Score; PHQ-A: Patient Health Questionnaire for Adolescents; CTQ: Childhood Trauma Questionnaire.

|  | **Low Risk**  **Mean (SD)** | | **High Risk**  **Mean (SD)** | | **MDD**  **Mean (SD)** | |
| --- | --- | --- | --- | --- | --- | --- |
|  | Males | Females | Males | Females | Males | Females |
| Age | 15.3 (0.8) | 15.4 (0.9) | 15.8 (0.9) | 15.7 (0.7) | 15.8 (0.8) | 15.8 (0.7) |
| Body Mass Index | 22  (5.6) | 23.2 (5.3) | 22.9 (5.0) | 21.8 (4.6) | 22.8 (4.2) | 22.6 (3.5) |
| PHQ - A | 2.80 (1.5) | 2.84 (1.5) | 3.80 (1.4) | 4.12 (1.7) | 17.84 (4.3) | 19.80 (4.4) |
| CTQ | 28.96 (3.0) | 29.36 (3.7) | 37.96 (9.2) | 38.29 (7.3) | 51.92 (12.5) | 51.20 (14.0) |
|  | **Low Risk  n (%)** | | **High Risk  n(%)** | | **MDD  n(%)** | |
|  | Males | Females | Males | Females | Males | Females |
| Skin color, non-white | 14 (56%) | 8  (32%) | 9 (36%) | 17 (60%) | 14 (56%) | 10  (40%) |
| Meets friends | 24 (96%) | 25 (100%) | 21 (84%) | 19 (76%) | 20 (80%) | 10  (40%) |
| School Failure | 0  (0%) | 0  (0%) | 15 (60%) | 14 (56%) | 13 (52%) | 12  (48%) |
| Ran away | 1  (4%) | 0  (0%) | 1  (4%) | 2  (8%) | 10 (40%) | 3 (12%) |
| Any drug use | 16 (64%) | 13 (52%) | 21 (84%) | 23 (92%) | 22 (88%) | 25 (100%) |
| Fights | 0  (0%) | 0  (0%) | 15 (60%) | 5  (20%) | 17 (60%) | 10  (40%) |
| Relationship with father (mean, SD) | 4.68 (0.47) | 4.36 (0.99) | 3.04 (1.13) | 1.92 (1.03) | 2.36 (1.38) | 1.64 (0.81) |
| Relationship with mother (mean, SD) | 4.92 (0.27) | 4.64 (0.70) | 4.08 (0.99) | 3.76 (1.01) | 3.12 (1.16) | 3.16 (1.14) |
| Relationship between parents (mean, SD) | 4.52 (0.71) | 3.84 (1.28) | 2.56 (1.22) | 2.20 (1.22) | 2.08 (1.18) | 1.80 (0.86) |
| Childhood Maltreatment - None | 25 (100%) | 25 (100%) | 1  (4%) | 0  (0%) | 0  (0%) | 0  (0%) |
| Childhood Maltreatment - Probable | 0  (0%) | 0  (0%) | 6 (24%) | 6  (24%) | 4  (16%) | 0  (0%) |
| Childhood Maltreatment - Severe | 0  (0%) | 0  (0%) | 18 (72%) | 19 (76%) | 21 (84%) | 25 (100%) |

## Table S2. Numerosity of the transcripts, before and after applying the filters.

|  | **MDD vs HR** | | | **HR vs LR** | | | **MDD vs LR** | | |
| --- | --- | --- | --- | --- | --- | --- | --- | --- | --- |
| **Transcript counts** | **ALL** | **Female** | **Male** | **ALL** | **Female** | **Male** | **ALL** | **Female** | **Male** |
| Total | 60230 | 60230 | 60230 | 60230 | 60230 | 60230 | 60230 | 60230 | 60230 |
| After filtering rows <10 reads | 30462 | 27630 | 27940 | 30321 | 27630 | 27940 | 30383 | 27485 | 27946 |
| After filtering NA genes | 21356 | 20092 | 20235 | 21296 | 20092 | 20235 | 21339 | 20019 | 20237 |
| After filtering non protein-coding transcripts | 16802 | 16252 | 16337 | 16786 | 16252 | 16337 | 16774 | 16249 | 16334 |

## Table S3. 40 pathways MDD vs HR (p-value < 0.05).

The z-score is a statistical measure of how closely the actual expression pattern of the DE transcripts in the uploaded datasets compare to the pattern that is expected based on the literature for each specific pathway identified. The possible results that IPA provides in terms of z-score are: i) z-score > 2 meaning a predicted activation of that pathway (an up-regulation), ii) z-score < -2 meaning a predicted inactivation (a down-regulation); iii) no z-score, meaning that the software was not able to identify whether the pathway was activated or inactivated based on the DE transcripts provided (Kramer, Green, Pollard, & Tugendreich, 2014).

| **Ingenuity Canonical Pathways** | **p-value** | **z-score** | **Molecules** |
| --- | --- | --- | --- |
| Interferon alpha/beta signaling | <0.001 | 3.873 | IFI27,IFI6,IFIT1,IFIT2,IFIT3,IFIT5,IFITM3,ISG15,MX1,OAS1,OAS2,OAS3,OASL,RSAD2,USP18 |
| Role of Hypercytokinemia/hyperchemokinemia in the Pathogenesis of Influenza | <0.001 | 3.464 | CCL2,CXCL10,EIF2AK2,IFIT2,IFIT3,ISG15,MX1,OAS1,OAS2,OAS3,RSAD2,TLR3 |
| Interferon Signaling | <0.001 | 2.646 | IFI6,IFIT1,IFIT3,IFITM3,ISG15,MX1,OAS1 |
| OAS antiviral response | <0.001 | 2 | OAS1,OAS2,OAS3,OASL |
| Role of Pattern Recognition Receptors in Recognition of Bacteria and Viruses | <0.001 | 2.449 | C1QA,C1QB,C1QC,EDA,EIF2AK2,IFIH1,OAS1,OAS2,OAS3,TLR3,TNFSF10 |
| Complement System | 0.002 | 2 | C1QA,C1QB,C1QC,C4BPA |
| Agranulocyte Adhesion and Diapedesis | 0.003 |  | CCL2,CCL25,CCL8,CLDN12,CXCL10,GNAI1,MMP23B,MMP8,MYH11 |
| Binding and Uptake of Ligands by Scavenger Receptors | 0.003 | 1 | COL1A2,COLEC12,HP,MSR1 |
| Granulocyte Adhesion and Diapedesis | 0.005 |  | CCL2,CCL25,CCL8,CLDN12,CXCL10,GNAI1,MMP23B,MMP8 |
| ISGylation Signaling Pathway | 0.005 | 2.449 | EIF2AK2,HERC5,IFIH1,ISG15,TLR3,USP18 |
| Interferon gamma signaling | 0.005 | 2.449 | MT2A,OAS1,OAS2,OAS3,OASL,TRIM6 |
| Complement cascade | 0.007 | 1 | C1QA,C1QB,C1QC,C4BPA |
| Extracellular matrix organization | 0.008 | 2.449 | AGRN,COL1A2,COL4A4,DCN,ITGA8,SERPINE1 |
| Pathogen Induced Cytokine Storm Signaling Pathway | 0.008 | 2.887 | CCL2,CCL25,CCL8,COL15A1,COL1A2,COL4A4,CXCL10,DHX58,EDA,IFIH1,TLR3,TNFSF10 |
| Role of MAPK Signaling in Inhibiting the Pathogenesis of Influenza | 0.008 | 0.447 | CCL2,CXCL10,EIF2AK2,PLA2G2D,PLA2G4C |
| ISG15 antiviral mechanism | 0.010 | 2.236 | EIF2AK2,HERC5,IFIT1,ISG15,MX1 |
| Activation of Matrix Metalloproteinases | 0.012 |  | MMP8,PLG,SPOCK3 |
| Estrogen-mediated S-phase Entry | 0.012 |  | CCNA1,CCNE2,CDC25A |
| Atherosclerosis Signaling | 0.012 |  | CCL2,COL1A2,LPL,MSR1,PLA2G2D,PLA2G4C |
| Calcium Transport I | 0.014 |  | ATP2A1,ATP2B3 |
| Integrin cell surface interactions | 0.014 | 1.342 | AGRN,COL1A2,COL4A4,ITGA8,SPP1 |
| Activation of IRF by Cytosolic Pattern Recognition Receptors | 0.014 | 0 | DHX58,IFIH1,IFIT2,ISG15 |
| Collagen degradation | 0.017 | 1 | COL15A1,COL1A2,COL4A4,MMP8 |
| G alpha (i) signalling events | 0.018 | 1.667 | CCL25,CXCL10,DRD3,GNAI1,GPER1,GRM7,RGS13,RGS16,SUCNR1 |
| Threonine Degradation II | 0.019 |  | GCAT |
| Degradation of the extracellular matrix | 0.019 |  | DCN,MMP8,PLG,SPP1 |
| Dissolution of Fibrin Clot | 0.023 |  | PLG,SERPINE1 |
| Triacylglycerol Degradation | 0.025 |  | LPL,NDST3,TNFAIP6 |
| Role of PKR in Interferon Induction and Antiviral Response | 0.025 |  | COLEC12,EIF2AK2,IFIH1,MARCO,MSR1,TLR3 |
| Acute Phase Response Signaling | 0.030 |  | C1QA,C1QB,C1QC,C4BPA,HP,PLG,SERPINE1 |
| Eukaryotic Translation Elongation | 0.030 | -2.236 | EEF1B2,RPL11,RPL34,RPL36A,RPL9 |
| Class A/1 (Rhodopsin-like receptors) | 0.041 | 0.333 | CCL2,CCL25,CXCL10,DRD3,GPER1,LPAR4,SUCNR1,TAC3,TACR3 |
| Collagen chain trimerization | 0.042 |  | COL15A1,COL1A2,COL4A4 |
| Hepatic Fibrosis / Hepatic Stellate Cell Activation | 0.045 |  | BAMBI,CCL2,COL15A1,COL1A2,COL4A4,MYH11,SERPINE1 |
| FXR/RXR Activation | 0.046 | -1.633 | EDA,GSTA4,GSTM5,LPL,PPARG,TNFSF10 |
| HEY1 Signaling Pathway | 0.046 |  | BMP6,HEY1,MMP23B,MMP8,MYH11,NEUROD4 |
| Role of MAPK Signaling in the Pathogenesis of Influenza | 0.046 |  | CCL2,CXCL10,PLA2G2D,PLA2G4C |
| Coronavirus Pathogenesis Pathway | 0.047 | -1.134 | CCL2,CCNE2,OAS1,OAS2,OAS3,SERPINE1,TLR3 |
| Oxytocin Signaling Pathway | 0.047 |  | ATP2B3,GNAI1,KCNJ8,LPL,MYH11,PLA2G2D,PLA2G4C,PPARG,SHC3 |
| Interleukin-10 signaling | 0.048 |  | CCL2,CD80,CXCL10 |

## Table S4. 80 pathways MDD vs LR (p-value < 0.05)

| **Ingenuity Canonical Pathways** | **p-value** | **z-score** | **Molecules** |
| --- | --- | --- | --- |
| Interferon alpha/beta signaling | <0.001 | 3.742 | IFI6,IFIT1,IFIT2,IFIT3,IFIT5,IRF7,ISG15,MX1,MX2,OAS2,OAS3,RSAD2,USP18,XAF1 |
| Role of Hypercytokinemia/hyperchemokinemia in the Pathogenesis of Influenza | <0.001 | 3.317 | CCL2,EIF2AK2,IFIT2,IFIT3,IRF7,ISG15,MX1,OAS2,OAS3,RIGI,RSAD2 |
| Glutamate Receptor Signaling | <0.001 | -1.633 | GRIA1,GRIA2,GRIA3,GRIN1,GRIN3B,GRIP1,GRM6,GRM7,HOMER1,SLC17A7,SLC1A2 |
| Glutamate binding, activation of AMPA receptors and synaptic plasticity | <0.001 | -2.828 | CAMK2A,EPB41L1,GRIA1,GRIA2,GRIA3,GRIP1,GRIP2,TSPAN7 |
| Calcium Signaling | <0.001 | -1.508 | ACTC1,ATP2B2,CACNA1E,CAMK2A,CASQ1,CHRNB2,GRIA1,GRIA2,GRIA3,GRIN1,GRIN3B,MYH10,MYH11,MYO1A,RYR1,TNNI3,TNNT1,TP63 |
| Neurovascular Coupling Signaling Pathway | <0.001 | -2.183 | CACNA1E,GABRA3,GABRD,GABRR2,GAD1,GRIA1,GRIA2,GRIA3,GRIN1,GRIN3B,KCNJ10,KCNJ2,KCNMA1,MAPT,NPR1,RYR1,SLC1A2 |
| Synaptogenesis Signaling Pathway | <0.001 | -2.4 | ADCY2,AFDN,CAMK2A,EFNB2,EPHA5,EPHA7,GRIA1,GRIA2,GRIA3,GRIN1,GRIN3B,GRM6,GRM7,MAPT,NRXN2,RASD2,SHC3,STXBP1,STXBP4,STXBP6,SYN3,WASF1 |
| Activation of NMDA receptors and postsynaptic events | <0.001 | -2.828 | CAMK2A,ERBB4,GRIA1,GRIA2,GRIA3,GRIN1,MAPT,PPM1E,TUBB3,TUBB8B |
| Neurexins and neuroligins | <0.001 | -2.828 | APBA1,DLGAP1,DLGAP3,EPB41L1,GRIN1,HOMER1,NRXN2,STXBP1 |
| Assembly and cell surface presentation of NMDA receptors | 0.001 | -1.633 | APBA1,CAMK2A,GRIN1,GRIN3B,TUBB3,TUBB8B |
| Neuropathic Pain Signaling in Dorsal Horn Neurons | 0.001 | -2.333 | CAMK2A,GRIA1,GRIA2,GRIA3,GRIN1,GRIN3B,GRM6,GRM7,KCNQ2 |
| Retinoid metabolism and transport | 0.001 | -0.447 | AGRN,GPC3,LPL,SDC1,SDC2 |
| OAS antiviral response | 0.002 |  | OAS2,OAS3,RIGI |
| Cardiac conduction | 0.002 | -1.897 | ATP1A3,ATP1B1,ATP2B2,CAMK2A,CASQ1,KCNJ2,NPR1,RYR1,SCN4B,TNNI3 |
| Interferon Signaling | 0.002 | 2.236 | IFI6,IFIT1,IFIT3,ISG15,MX1 |
| Role of Pattern Recognition Receptors in Recognition of Bacteria and Viruses | 0.002 | 2.449 | C1QB,C1QC,EDA,EIF2AK2,IFIH1,IRF7,OAS2,OAS3,RIGI,TNFSF10 |
| Synaptic adhesion-like molecules | 0.002 | -2 | GRIA1,GRIA3,GRIN1,PTPRF |
| Activation of IRF by Cytosolic Pattern Recognition Receptors | 0.003 | 1.633 | IFIH1,IFIT2,IRF7,ISG15,RIGI,ZBP1 |
| ISG15 antiviral mechanism | 0.003 | 2.646 | EIF2AK2,HERC5,IFIT1,ISG15,MX1,MX2,RIGI |
| Synaptic Long Term Depression | 0.003 | -1.897 | CACNA1E,CRHR1,GAD1,GRIA1,GRIA2,GRIA3,GRM6,GRM7,NPR1,PPP2R2C,RASD2,RYR1 |
| Neurotransmitter release cycle | 0.004 | -2.236 | APBA1,SLC17A7,SLC1A2,STXBP1,SYN3 |
| Kinesins | 0.004 | -1.633 | KIF4A,KIF5A,KIF9,KLC3,TUBB3,TUBB8B |
| Threonine catabolism | 0.004 |  | GCAT,SDS |
| HEY1 Signaling Pathway | 0.005 | 1 | BMP6,BMP8A,DLL3,E2F7,ERBB4,MMP8,MYH11,NTRK3,SMAD1,VEGFC |
| Tight Junction Signaling | 0.005 |  | ACTC1,ACTG2,AFDN,CRB3,MAGI2,MYH10,MYH11,MYO1A,NECTIN2,PPP2R2C,TJP1 |
| Dilated Cardiomyopathy Signaling Pathway | 0.005 | 0.707 | ACTC1,ACTG2,ADCY2,CACNA1E,CAMK2A,MYH10,MYH11,MYO1A,TNNI3,TNNT1 |
| Glycosaminoglycan metabolism | 0.005 | 0.378 | AGRN,B4GALT6,CSPG5,DCN,GPC3,SDC1,SDC2 |
| SNARE Signaling Pathway | 0.007 | -1.667 | ADCY2,CAMK2A,MYH10,MYH11,MYO1A,STXBP1,STXBP4,STXBP6,SYN3 |
| Synaptic Long Term Potentiation | 0.007 | -2.121 | CAMK2A,GRIA1,GRIA2,GRIA3,GRIN1,GRIN3B,GRM6,GRM7,RASD2 |
| Glutaminergic Receptor Signaling Pathway (Enhanced) | 0.007 | -1.5 | ADCY2,CACNA1E,CAMK2A,GABRA3,GABRD,GABRR2,GRIA1,GRIA2,GRIA3,GRIN1,GRIN3B,GRM6,GRM7,HOMER1,SCN4B,SLC1A2 |
| ISGylation Signaling Pathway | 0.009 | 1.89 | EIF2AK2,HERC5,IFIH1,IRF7,ISG15,RIGI,USP18 |
| CREB Signaling in Neurons | 0.009 | -1.46 | ADCY2,ADGRA3,ADGRG6,ADGRL3,BMP6,CACNA1E,CAMK2A,CRHR1,FFAR3,FGFR4,GPR176,GRIA1,GRIA2,GRIA3,GRIN1,GRM6,GRM7,HCAR2,HCAR3,HTR2B,NTRK3,RASD2,SHC3,TACR3 |
| GABAergic Receptor Signaling Pathway (Enhanced) | 0.009 | -1 | ADCY2,CACNA1E,GABRA3,GABRD,GABRR2,GAD1,GRIN1,GRIN3B,NRXN2 |
| Airway Pathology in Chronic Obstructive Pulmonary Disease | 0.009 |  | CCL2,EDA,ELANE,FGF17,LCN2,MMP8,TNFSF10 |
| Agrin Interactions at Neuromuscular Junction | 0.009 | -1 | ACTC1,ACTG2,AGRN,ERBB4,LAMA2,RASD2 |
| Amyotrophic Lateral Sclerosis Signaling | 0.010 | -0.378 | CACNA1E,GRIA1,GRIA2,GRIA3,GRIN1,GRIN3B,SLC1A2,VEGFC |
| Interferon gamma signaling | 0.010 | 1.89 | CAMK2A,IRF7,MT2A,OAS2,OAS3,TRIM22,TRIM6 |
| Cellular Effects of Sildenafil (Viagra) | 0.010 |  | ACTC1,ACTG2,ADCY2,CACNA1E,KCNQ2,MYH10,MYH11,MYO1A,NPR1 |
| Germ layer formation at gastrulation | 0.010 |  | FOXH1,MIXL1,SOX2 |
| Neurotransmitter receptors and postsynaptic signal transmission | 0.010 |  | GLRA2,GLRB |
| EPH-Ephrin signaling | 0.013 | -1.89 | EFNB2,EPHA5,EPHA7,GRIN1,MYH10,MYH11,SDC2 |
| Gap Junction Signaling | 0.014 | -0.775 | ACTC1,ACTG2,ADCY2,GAD1,GRIA1,GRIA2,GRIA3,HTR2B,KCNQ2,LRP5,NPR1,RASD2,SMAD1,TJP1,TUBB3 |
| mRNA Editing | 0.014 |  | APOBEC3A,APOBEC3B |
| Extracellular matrix organization | 0.015 | 1.89 | AGRN,CEACAM8,DCN,ITGA8,ITGA9,LAMA2,TNR |
| Endocannabinoid Neuronal Synapse Pathway | 0.016 | -0.707 | ADCY2,CACNA1E,FAAH,GRIA1,GRIA2,GRIA3,GRIN1,GRIN3B,MAPK4 |
| L1CAM interactions | 0.016 | -0.707 | ANK2,ITGA9,KCNQ2,KIF4A,NRCAM,SCN4B,TUBB3,TUBB8B |
| GABA receptor activation | 0.017 | -0.447 | ADCY2,GABRA3,GABRR2,KCNJ10,KCNJ2 |
| Potassium Channels | 0.018 | -1.134 | ABCC8,KCNAB1,KCNC1,KCNJ10,KCNJ2,KCNMA1,KCNQ2 |
| Ephrin Receptor Signaling | 0.019 | -1 | EFNB2,EPHA5,EPHA7,GRIN1,GRIN3B,ITGA8,ITGA9,PTPN13,RASD2,SDC2,VEGFC |
| Gustation Pathway | 0.019 |  | ABCC8,ADCY2,ASIC2,CACNA1E,GABRA3,GABRD,GABRR2,KCNQ2,LPL,SCN4B |
| Carboxyterminal post-translational modifications of tubulin | 0.019 | -1 | AGBL2,TTLL10,TUBB3,TUBB8B |
| Complement cascade | 0.021 | 1 | C1QB,C1QC,C4BPA,ELANE |
| Transcriptional Regulation by MECP2 | 0.022 | -1 | CAMK2A,GAD1,GRIA2,PPARG,SOX2 |
| Salvage Pathways of Pyrimidine Deoxyribonucleotides | 0.023 |  | APOBEC3A,APOBEC3B |
| Glycine Betaine Degradation | 0.023 |  | SARDH,SDS |
| Glutathione-mediated Detoxification | 0.024 |  | GGH,GSTA1,GSTA4 |
| Crosstalk between Dendritic Cells and Natural Killer Cells | 0.025 | 1 | ACTC1,ACTG2,CAMK2A,CD80,NECTIN2,TNFSF10 |
| Epithelial Adherens Junction Signaling | 0.025 | 0.333 | AFDN,MAGI1,MAGI2,MYH10,NECTIN2,PPP2R2C,RASD2,SFN,WASF1 |
| Adrenomedullin signaling pathway | 0.025 | -1.414 | ADCY2,ADM,GAD1,KCNQ2,MAPK4,NPR1,PPARG,RASD2,SHC3,TFAP2A |
| Sleep NREM Signaling Pathway | 0.026 | -0.378 | ADCY2,CAMK2A,GABRA3,GABRD,GABRR2,RASD2,SFN |
| Sensory processing of sound by inner hair cells of the cochlea | 0.027 | -1.342 | ATP2B2,BSN,EPB41L1,KCNMA1,SYP |
| cAMP-mediated signaling | 0.027 | -0.302 | ADCY2,CAMK2A,CRHR1,FFAR3,GRM6,GRM7,HCAR2,HCAR3,PDE8B,RGS4,TULP2 |
| Glutamate Removal from Folates | 0.028 |  | GGH |
| Threonine Degradation II | 0.028 |  | GCAT |
| Osteoarthritis Pathway | 0.029 |  | C1QTNF4,CASP5,CASQ1,DCN,IL18RAP,ITGA8,ITGA9,PPARG,PRG4,SMAD1,VEGFC |
| Gαi Signaling | 0.030 | 0.816 | ADCY2,FFAR3,GRM6,GRM7,HCAR2,RASD2,RGS4,SHC3 |
| Airway Inflammation in Asthma | 0.033 |  | CCL2,ELANE,RNASE2 |
| DDX58/IFIH1-mediated induction of interferon-alpha/beta | 0.034 | 2.236 | HERC5,IFIH1,IRF7,ISG15,RIGI |
| RHO GTPases activate KTN1 | 0.035 |  | KIF5A,KLC3 |
| Neuroinflammation Signaling Pathway | 0.035 | 0.333 | CCL2,CD80,GABRA3,GABRD,GABRR2,GAD1,GRIA1,GRIN1,GRIN3B,IRF7,MAPK4,MAPT,SLC1A2 |
| Pyroptosis | 0.036 |  | CASP5,ELANE,TP63 |
| Ion channel transport | 0.039 | -2.333 | ASIC2,ATP1A3,ATP1B1,ATP2B2,ATP8B4,CAMK2A,CASQ1,RYR1,TTYH1 |
| Actin Cytoskeleton Signaling | 0.039 | -1.633 | ACTC1,ACTG2,FGF17,ITGA8,ITGA9,MYH10,MYH11,MYO1A,PFN2,RASD2,WASF1 |
| RHO GTPases activate PKNs | 0.040 |  | MYH10,MYH11,SFN |
| Complement System | 0.040 |  | C1QB,C1QC,C4BPA |
| FXR/RXR Activation | 0.041 | -1.414 | EDA,FGFR4,GSTA1,GSTA4,LPL,PPARG,SDC1,TNFSF10 |
| Circadian Rhythm Signaling | 0.041 |  | ADCY2,CACNA1E,CAMK2A,GAD1,GRIA1,GRIA2,GRIA3,GRIN1,GRIN3B,NPR1,RASD2,RYR1 |
| Oxytocin Signaling Pathway | 0.046 | -1.155 | ATP2B2,CACNA1E,GAD1,LPL,MAPK4,MYH10,MYH11,MYO1A,NPR1,PPARG,RASD2,SHC3 |
| Inhibition of Matrix Metalloproteases | 0.048 |  | MMP8,SDC1,SDC2 |
| GABA Receptor Signaling | 0.048 |  | ADCY2,CACNA1E,GABRA3,GABRD,GABRR2,GAD1,KCNQ2 |

## Table S5. 48 pathways HR vs LR (p-value < 0.05)

| **Ingenuity Canonical Pathways** | **p-value** | **z-score** | **Molecules** |
| --- | --- | --- | --- |
| Pulmonary Fibrosis Idiopathic Signaling Pathway | <0.001 | -1.508 | ACTG2,AREG,CAV1,COL16A1,FGF9,FGFR4,ITGAV,MMP1,MMP21,MRAS,PDGFC,PLG |
| L1CAM interactions | <0.001 | -1.89 | ANK2,ITGAV,KCNQ2,NRCAM,SCN2A,SCN4B,TUBB3 |
| Axonal Guidance Signaling | 0.001 |  | DOCK1,GLI3,ITGAV,MMP1,MMP21,MRAS,MYL11,NFATC4,NTN5,NTRK2,PDGFC,RHOD,SRGAP1,TUBB3 |
| Cellular Effects of Sildenafil (Viagra) | 0.001 |  | ACTG2,GUCY1A1,KCNQ2,MYL11,MYO18B,PDE1A,PRKG2 |
| Actin Cytoskeleton Signaling | 0.001 | -2.236 | ACTG2,DOCK1,FGF9,ITGAV,MRAS,MYL11,MYO18B,PDGFC,TLN2 |
| Nitric Oxide Signaling in the Cardiovascular System | 0.001 | -1.342 | CAV1,GUCY1A1,PDE1A,PDGFC,PRKG2,RYR2 |
| Cell surface interactions at the vascular wall | 0.003 | -1.633 | CAV1,GRB14,ITGAV,MMP1,SLC7A9,VPREB1 |
| Bladder Cancer Signaling | 0.005 |  | FGF9,MMP1,MMP21,MRAS,PDGFC |
| Neurovascular Coupling Signaling Pathway | 0.007 | -1.134 | ENTPD3,GRIN3B,GRM5,GUCY1A1,KCNJ10,PRKG2,RYR2 |
| Paxillin Signaling | 0.007 | -2 | ACTG2,DOCK1,ITGAV,MRAS,TLN2 |
| Glioma Invasiveness Signaling | 0.009 |  | ITGAV,MRAS,PLG,RHOD |
| Integrin Signaling | 0.009 | -1.89 | ACTG2,CAV1,DOCK1,ITGAV,MRAS,RHOD,TLN2 |
| Gαs Signaling | 0.009 | -1 | GLP1R,GUCY1A1,MRAS,PTH1R,RYR2 |
| Sensory perception of taste | 0.009 |  | SCN2A,SCN4B,SCNN1D |
| Dissolution of Fibrin Clot | 0.009 |  | PLG,SERPINE2 |
| Smooth Muscle Contraction | 0.011 |  | ACTG2,GUCY1A1,MYL11 |
| Assembly and cell surface presentation of NMDA receptors | 0.011 |  | APBA1,GRIN3B,TUBB3 |
| Platelet homeostasis | 0.014 | -2 | GUCY1A1,PDE1A,PRKG2,SLC8A2 |
| Role of JAK2 in Hormone-like Cytokine Signaling | 0.017 |  | PDGFC,POMC,PRL |
| Gap Junction Signaling | 0.019 | -2.121 | ACTG2,CAV1,GUCY1A1,KCNQ2,LRP5,MRAS,PRKG2,TUBB3 |
| ID1 Signaling Pathway | 0.021 | -1.633 | CAV1,CHRFAM7A,FGFR4,MRAS,PDGFC,TFAP2A |
| Semaphorin Neuronal Repulsive Signaling Pathway | 0.022 | -0.447 | DPYSL4,GUCY1A1,ITGAV,MYL11,PRKG2 |
| Choline Degradation I | 0.023 |  | CHDH |
| Thyroid Hormone Biosynthesis | 0.023 |  | TPO |
| Cysteine Biosynthesis/Homocysteine Degradation | 0.023 |  | CBS/LOC102724560 |
| Colorectal Cancer Metastasis Signaling | 0.025 | -1.342 | GUCY1A1,LRP5,MMP1,MMP21,MRAS,PDGFC,RHOD |
| Neuropathic Pain Signaling in Dorsal Horn Neurons | 0.025 | -1 | GRIN3B,GRM5,KCNQ2,NTRK2 |
| Calcium Signaling | 0.026 | 0.447 | CHRFAM7A,GRIN3B,MYO18B,NFATC4,RYR2,SLC8A2 |
| Osteoarthritis Pathway | 0.033 | -1 | GLI3,ITGAV,ITLN1,MMP1,PDGFC,PTH1R |
| Triglyceride metabolism | 0.033 |  | CAV1,PLIN1 |
| Regulation of Actin-based Motility by Rho | 0.034 |  | ACTG2,ITGAV,MYL11,RHOD |
| RHOGDI Signaling | 0.034 | 1 | ACTG2,ITGAV,MRAS,MYL11,MYO18B,RHOD |
| Tumor Microenvironment Pathway | 0.035 | -2.236 | FGF9,MMP1,MMP21,MRAS,PDGFC |
| Gαq Signaling | 0.035 | 0.447 | ARHGEF25,GRM5,MRAS,NFATC4,RHOD |
| Agranulocyte Adhesion and Diapedesis | 0.035 |  | ACTG2,CCL25,MMP1,MMP21,MYO18B |
| Activation of Matrix Metalloproteinases | 0.038 |  | MMP1,PLG |
| G alpha (s) signalling events | 0.039 | -2 | GLP1R,PDE1A,POMC,PTH1R |
| CXCR4 Signaling | 0.039 | -1 | DOCK1,GUCY1A1,MRAS,MYL11,RHOD |
| Germ Cell-Sertoli Cell Junction Signaling | 0.039 |  | ACTG2,MRAS,NECTIN2,RHOD,TUBB3 |
| PAK Signaling | 0.041 |  | ITGAV,MRAS,MYL11,PDGFC |
| Role of JAK family kinases in IL-6-type Cytokine Signaling | 0.042 |  | MMP1,PDGFC,POMC |
| Autism Signaling Pathway | 0.043 | -0.378 | CBS/LOC102724560,GRIN3B,GRM5,LRP5,MRAS,NTRK2,SLC6A8 |
| Cardiac β-adrenergic Signaling | 0.046 |  | GUCY1A1,MRAS,PDE1A,RYR2,SLC8A2 |
| Oxytocin in Spinal Neurons Signaling Pathway | 0.046 |  | GUCY1A1,PRKG2 |
| Inhibition of Matrix Metalloproteases | 0.046 |  | MMP1,MMP21 |
| Sperm Motility | 0.047 | -2 | FGFR4,GUCY1A1,MRAS,NTRK2,PDE1A,PRKG2 |
| Gustation Pathway | 0.047 |  | GLP1R,GUCY1A1,KCNQ2,SCN2A,SCN4B |
| Cardiac conduction | 0.049 |  | RYR2,SCN2A,SCN4B,SLC8A2 |

## Table S6. 73 pathways females MDD vs females HR (p-value < 0.05)

| **Ingenuity Canonical Pathways** | **p-value** | **z-score** | **Molecules** |
| --- | --- | --- | --- |
| Interferon alpha/beta signaling | <0.001 | 4.359 | BST2,IFI27,IFI6,IFIT1,IFIT2,IFIT3,IFIT5,IFITM3,IRF7,ISG15,MX1,MX2,OAS1,OAS2,OAS3,OASL,RSAD2,STAT2,USP18 |
| Role of Hypercytokinemia/hyperchemokinemia in the Pathogenesis of Influenza | <0.001 | 4.243 | CCL2,CCL3,CXCL10,CXCL8,EIF2AK2,IFIT2,IFIT3,IL1RN,IRF7,ISG15,MX1,OAS1,OAS2,OAS3,RIGI,RSAD2,STAT2,TLR3 |
| Interferon Signaling | <0.001 | 3 | IFI6,IFIT1,IFIT3,IFITM3,ISG15,JAK2,MX1,OAS1,STAT2 |
| OAS antiviral response | <0.001 | 2.236 | OAS1,OAS2,OAS3,OASL,RIGI |
| Role of Pattern Recognition Receptors in Recognition of Bacteria and Viruses | <0.001 | 2.646 | C1QB,CXCL8,EIF2AK2,IFIH1,IRF7,OAS1,OAS2,OAS3,OSM,PIK3C2A,RIGI,TLR3,TNFSF10,TNFSF13B |
| Systemic Lupus Erythematosus in B Cell Signaling Pathway | <0.001 | 2.668 | CD19,CD72,CD79A,CXCL8,FCGR2B,IFIH1,IFIT2,IFIT3,IRF7,ISG15,JAK2,OSM,PIK3C2A,STAT2,TLR3,TNFSF10,TNFSF13B |
| Interferon gamma signaling | <0.001 | 3.162 | IRF7,JAK2,MT2A,OAS1,OAS2,OAS3,OASL,TRIM22,TRIM5,TRIM6 |
| Agranulocyte Adhesion and Diapedesis | <0.001 |  | CCL2,CCL3,CCL8,CCR9,CLDN23,CXCL10,CXCL8,GNAI1,IL1RN,MMP24,MMP8,MYH11,XCL1 |
| Granulocyte Adhesion and Diapedesis | <0.001 |  | CCL2,CCL3,CCL8,CCR9,CLDN23,CXCL10,CXCL8,GNAI1,IL1RN,MMP24,MMP8,XCL1 |
| Activation of IRF by Cytosolic Pattern Recognition Receptors | <0.001 | 1.134 | DHX58,IFIH1,IFIT2,IRF7,ISG15,RIGI,STAT2 |
| ISGylation Signaling Pathway | <0.001 | 2.333 | EIF2AK2,HERC5,IFIH1,IRF7,ISG15,RIGI,STAT2,TLR3,USP18 |
| Role of Osteoblasts in Rheumatoid Arthritis Signaling Pathway | <0.001 | 0.535 | APC2,CXCL8,FZD5,JAK2,LRP6,MMP24,MMP8,OSM,PIK3C2A,STAT2,TNFSF10,TNFSF13B,WNT10A,WNT5B |
| Pathogen Induced Cytokine Storm Signaling Pathway | <0.001 | 3.638 | CCL2,CCL3,CCL8,COL15A1,CXCL10,CXCL8,DHX58,IFIH1,IL1RN,IRF7,JAK2,OSM,RIGI,TLR3,TNFSF10,TNFSF13B,XCL1 |
| Airway Pathology in Chronic Obstructive Pulmonary Disease | 0.001 |  | CCL2,CXCL8,LCN12,LCN2,MMP8,OSM,TNFSF10,TNFSF13B |
| Communication between Innate and Adaptive Immune Cells | 0.001 |  | CCL3,CD79A,CXCL10,CXCL8,IL1RN,TLR3,TNFSF13B |
| Role of MAPK Signaling in Inhibiting the Pathogenesis of Influenza | 0.001 | 1.89 | CCL2,CXCL10,CXCL8,EIF2AK2,PLA2G4A,PLA2G4C,PLA2G7 |
| Salvage Pathways of Pyrimidine Deoxyribonucleotides | 0.001 |  | APOBEC3A,APOBEC3B,TYMP |
| TREM1 Signaling | 0.001 | 2.646 | CASP5,CCL2,CCL3,CXCL8,FCGR2B,JAK2,TLR3 |
| ISG15 antiviral mechanism | 0.002 | 2.646 | EIF2AK2,HERC5,IFIT1,ISG15,MX1,MX2,RIGI |
| Atherosclerosis Signaling | 0.002 |  | CCL2,CXCL8,IL1RN,MSR1,PLA2G4A,PLA2G4C,PLA2G7,TPSAB1/TPSB2 |
| Role of PKR in Interferon Induction and Antiviral Response | 0.002 | 1.633 | CASP5,EIF2AK2,IFIH1,IL24,MARCO,MSR1,RIGI,STAT2,TLR3 |
| Role of Cytokines in Mediating Communication between Immune Cells | 0.002 |  | CXCL8,IL1RN,IL24,IL27 |
| Coronavirus Pathogenesis Pathway | 0.003 | -1.508 | BST2,CCL2,CXCL8,IRF7,OAS1,OAS2,OAS3,RIGI,SERPINE1,STAT2,TLR3 |
| Interleukin-10 signaling | 0.004 | 2.236 | CCL2,CCL3,CXCL10,CXCL8,IL1RN |
| IL-17 Signaling | 0.004 | 3 | CCL2,CXCL8,IL17RC,JAK2,LCN2,OSM,PIK3C2A,TNFSF10,TNFSF13B |
| Differential Regulation of Cytokine Production in Intestinal Epithelial Cells by IL-17A and IL-17F | 0.005 |  | CCL2,CCL3,LCN2 |
| Role of RIG1-like Receptors in Antiviral Innate Immunity | 0.006 | 1 | DHX58,IFIH1,IRF7,RIGI |
| Role of Macrophages, Fibroblasts and Endothelial Cells in Rheumatoid Arthritis | 0.008 |  | APC2,CCL2,CXCL8,FZD5,IL17RC,IL1RN,JAK2,LRP6,OSM,PIK3C2A,TLR3,TNFSF13B,WNT10A,WNT5B |
| mRNA Editing | 0.011 |  | APOBEC3A,APOBEC3B |
| Interleukin-20 family signaling | 0.011 |  | IL24,JAK2,STAT2 |
| Crosstalk between Dendritic Cells and Natural Killer Cells | 0.013 | 2 | IL15RA,IL3RA,KIR3DL1,TLN2,TLR3,TNFSF10 |
| CDX Gastrointestinal Cancer Signaling Pathway | 0.013 | -1.667 | CXCL8,FZD5,JAK2,OSM,PIK3C2A,TNFSF10,TNFSF13B,WNT10A,WNT5B |
| Retinoate Biosynthesis I | 0.013 |  | ALDH1A1,ALDH8A1,RDH10 |
| Multiple Sclerosis Signaling Pathway | 0.013 | 3.162 | C1QB,CXCL8,IL17RC,OSM,PARP12,PARP9,SLC8A2,TLR3,TNFSF10,TNFSF13B |
| Autism Signaling Pathway | 0.014 | 2.496 | ALDH1A1,ALDH8A1,CCL2,CXCL8,FZD5,IL1RN,LRP6,OSM,PIK3C2A,TNFSF10,TNFSF13B,WNT10A,WNT5B |
| Complement cascade | 0.014 |  | C1QB,C1R,C4BPA,CD19 |
| Interleukin-6 family signaling | 0.015 |  | IL31RA,JAK2,OSM |
| Basal Cell Carcinoma Signaling | 0.015 | 1 | APC2,FZD5,KIF7,WNT10A,WNT5B |
| Role of NANOG in Mammalian Embryonic Stem Cell Pluripotency | 0.017 |  | APC2,FZD5,JAK2,PIK3C2A,TCL1A,WNT10A,WNT5B |
| Pathogenesis of Multiple Sclerosis | 0.019 |  | CCL3,CXCL10 |
| Airway Inflammation in Asthma | 0.019 |  | CCL2,CXCL8,OSM |
| IL-17A Signaling in Gastric Cells | 0.019 |  | CXCL10,CXCL8,IL17RC |
| MSP-RON Signaling Pathway | 0.019 |  | CCL2,IL3RA,JAK2,PIK3C2A |
| Role of IL-17F in Allergic Inflammatory Airway Diseases | 0.019 |  | CCL2,CXCL10,CXCL8,IL17RC |
| DDX58/IFIH1-mediated induction of interferon-alpha/beta | 0.020 | 2.236 | HERC5,IFIH1,IRF7,ISG15,RIGI |
| Macrophage Classical Activation Signaling Pathway | 0.021 | 2.828 | CXCL10,CXCL8,JAK2,OSM,PARP9,STAT2,TNFSF10,TNFSF13B |
| Role of Chondrocytes in Rheumatoid Arthritis Signaling Pathway | 0.023 | 1.89 | CCL2,CXCL8,IL17RC,IL1RN,JAK2,MMP24,MMP8 |
| Role of MAPK Signaling in the Pathogenesis of Influenza | 0.023 |  | CCL2,CXCL10,PLA2G4A,PLA2G4C,PLA2G7 |
| Activation of Matrix Metalloproteinases | 0.024 |  | MMP24,MMP8,TPSAB1/TPSB2 |
| B Cell Development | 0.024 |  | CD19,CD79A,RAG1 |
| NOD1/2 Signaling Pathway | 0.024 | 2.121 | CCL2,CXCL8,IRF7,OSM,RIGI,TLR3,TNFSF10,TNFSF13B |
| Cytosolic sensors of pathogen-associated DNA | 0.024 | 2 | IFI16,IRF7,TLR3,TREX1 |
| Role of IL-17A in Psoriasis | 0.028 |  | CXCL8,IL17RC |
| Acute Phase Response Signaling | 0.030 | 2.236 | C1QB,C1R,C4BPA,HP,IL1RN,JAK2,OSM,SERPINE1 |
| Interleukin-4 and Interleukin-13 signaling | 0.032 | 0.816 | CCL2,CXCL8,JAK2,LCN2,OSM,POMC |
| TR/RXR Activation | 0.032 | -0.816 | ATP2A1,HP,PIK3C2A,PPARGC1A,STRBP,THRB |
| Cachexia Signaling Pathway | 0.034 | 3.051 | CASP5,CCL2,CXCL8,EIF2AK2,IL1RN,LCN2,OSM,PIK3C2A,POMC,PTH2R,STAT2,TNFSF10,TNFSF13B |
| Complement System | 0.035 |  | C1QB,C1R,C4BPA |
| FXR/RXR Activation | 0.035 | -2.646 | CXCL8,GSTA4,IL1RN,OSM,PPARGC1A,TNFSF10,TNFSF13B |
| Trafficking and processing of endosomal TLR | 0.038 |  | TLR3,UNC93B1 |
| Differential Regulation of Cytokine Production in Macrophages and T Helper Cells by IL-17A and IL-17F | 0.038 |  | CCL2,CCL3 |
| Folate Signaling Pathway | 0.038 |  | ADARB2,FOLR2,JAK2,OAS2 |
| Role of IL-17A in Arthritis | 0.038 |  | CCL2,CXCL8,IL17RC,PIK3C2A |
| PCP (Planar Cell Polarity) Pathway | 0.041 | 1 | FZD5,PRICKLE1,WNT10A,WNT5B |
| Role of WNT/GSK-3β Signaling in the Pathogenesis of Influenza | 0.043 | 1 | APC2,FZD5,WNT10A,WNT5B |
| Glucocorticoid Receptor Signaling | 0.043 |  | CCL2,CCL3,CXCL8,ESR1,HP,IL15RA,IL17RC,IL1RN,IL31RA,IL3RA,JAK2,MMP8,PIK3C2A,PLA2G4A,PLA2G4C,POMC,SERPINE1 |
| Adenosine Nucleotides Degradation II | 0.044 |  | ADARB2,NT5C3A |
| Binding and Uptake of Ligands by Scavenger Receptors | 0.044 |  | HP,MSR1,SCARF1 |
| IL-17A Signaling in Fibroblasts | 0.046 | 2.236 | CCL2,CCL8,IL17RC,JAK2,LCN2 |
| CGAS-STING Signaling Pathway | 0.046 | 1.633 | CCL2,CXCL8,OSM,TNFSF10,TNFSF13B,TREX1 |
| Glycerol-3-phosphate Shuttle | 0.048 |  | GPD2 |
| Primary Immunodeficiency Signaling | 0.048 |  | CD19,CD79A,RAG1 |
| Prolactin receptor signaling | 0.049 |  | CSH1/CSH2,JAK2 |

## Table S7. 46 pathways females MDD vs females LR (p-value < 0.05)

| **Ingenuity Canonical Pathways** | **p-value** | **z-score** | **Molecules** |
| --- | --- | --- | --- |
| FAK Signaling | <0.001 | -0.18 | ADGRA3,ADGRF3,ADGRL3,ADRA1B,BCAR3,CCR9,COL18A1,CSF2RB,CXCR1,CXCR2,ERBB2,FFAR2,FZD5,GPR153,GPR20,GPR27,GPRC5C,HCAR2,HCAR3,HTR2B,IL3RA,ITGA7,ITGA9,ITGAV,LPAR2,MRAS,OXTR,PAK6,PDGFRB,S1PR3,TGFB2 |
| Phagosome Formation | <0.001 | -0.18 | ADGRA3,ADGRF3,ADGRL3,ADRA1B,CCR9,CXCR1,FCGR2A,FFAR2,FZD5,GPR153,GPR20,GPR27,GPRC5C,HCAR2,HCAR3,HTR2B,ITGA7,ITGA9,ITGAV,LIMK2,LPAR2,MARCKS,MRAS,MYD88,MYH10,MYO1A,OXTR,PAK6,S1PR3,TLR2,TTN |
| Molecular Mechanisms of Cancer | <0.001 | 1.151 | ADCY10,ADCY2,ADGRA3,ADGRF3,ADGRL3,ADRA1B,BMP6,CCR9,CDC25C,CSF2RB,CXCR1,CXCR2,FFAR2,FZD5,GNAQ,GPR153,GPR20,GPR27,GPRC5C,HCAR2,HCAR3,HTR2B,IL1B,IL3RA,ITGA7,ITGA9,ITGAV,LPAR2,LRP5,MRAS,OXTR,PAK6,PTCH1,RHOB,S1PR3,SMAD1,TGFB2 |
| CREB Signaling in Neurons | 0.001 | -0.392 | ADCY10,ADCY2,ADGRA3,ADGRF3,ADGRL3,ADRA1B,BMP6,CCR9,CXCR1,FFAR2,FGFR4,FZD5,GNAQ,GPR153,GPR20,GPR27,GPRC5C,HCAR2,HCAR3,HTR2B,LPAR2,MRAS,NTRK2,OXTR,PDGFRB,S1PR3,TGFB2 |
| STAT3 Pathway | 0.001 | -0.707 | BMP6,CSF2RB,CXCR1,CXCR2,FGFR4,IL1B,IL3RA,MRAS,NTRK2,PDGFRB,TGFB2 |
| BBSome Signaling Pathway | 0.001 | 0.218 | ADGRA3,ADGRF3,ADGRL3,ADRA1B,BBS7,CBX8,CCR9,CXCR1,FFAR2,FZD5,GPR153,GPR20,GPR27,GPRC5C,HCAR2,HCAR3,HTR2B,LPAR2,NTRK2,OXTR,PTCH1,S1PR3 |
| G-Protein Coupled Receptor Signaling | 0.002 | 0.928 | ADCY10,ADCY2,ADGRA3,ADGRF3,ADGRL3,ADRA1B,BORCS8-MEF2B,CCR9,CXCR1,CXCR2,FFAR2,FZD5,GNAQ,GPR153,GPR20,GPR27,GPRC5C,HCAR2,HCAR3,HCN4,HTR2B,LPAR2,MRAS,OXTR,PAK6,PDE7B,S1PR3,TTN,WWTR1 |
| Class A/1 (Rhodopsin-like receptors) | 0.002 | 1.291 | ACKR2,ADRA1B,CCL22,CCR9,CXCL16,CXCR1,CXCR2,FFAR2,GHRL,HCAR2,HCAR3,HTR2B,INSL3,LPAR2,OXTR |
| Breast Cancer Regulation by Stathmin1 | 0.002 | 0.6 | ADGRA3,ADGRF3,ADGRL3,ADRA1B,BMP6,CCR9,CXCR1,FFAR2,FZD5,GNAQ,GPR153,GPR20,GPR27,GPRC5C,HCAR2,HCAR3,HTR2B,LPAR2,MRAS,OXTR,PPP2R2C,PPP2R5A,S1PR3,TGFB2,TUBB3 |
| Extracellular matrix organization | 0.004 | -1.414 | COL18A1,COL4A2,ITGA7,ITGA9,ITGAV,LAMC1,NCAM1,TGFB2 |
| S100 Family Signaling Pathway | 0.005 |  | ABCB1,ADGRA3,ADGRF3,ADGRL3,ADRA1B,CCR9,CXCR1,ERBB2,ESR1,FCGR2A,FFAR2,FGFR4,FZD5,GPR153,GPR20,GPR27,GPRC5C,HCAR2,HCAR3,HTR2B,IL1B,LPAR2,MYD88,NTRK2,OXTR,S1PR3,SMAD1,TGFB2 |
| RHOGDI Signaling | 0.005 | 0.816 | CDH4,ESR1,GNAQ,GRIP1,ITGA7,ITGA9,ITGAV,LIMK2,MRAS,MYH10,MYO1A,PAK6,RHOB |
| Integrin cell surface interactions | 0.005 | -0.378 | COL16A1,COL18A1,COL4A2,ICAM4,ITGA7,ITGA9,ITGAV |
| Human Embryonic Stem Cell Pluripotency | 0.005 | -1.732 | BMP6,DPPA4,FGFR4,FZD5,INHBA,MRAS,NFYB,NTRK2,PDGFRB,SMAD1,TFCP2L1,TGFB2 |
| RHO GTPases activate PKNs | 0.007 |  | CDC25C,MYH10,RHOB,SFN |
| Agranulocyte Adhesion and Diapedesis | 0.008 |  | CCL22,CCR9,CLDN9,CXCL16,CXCR1,CXCR2,IL1B,IL1RN,MYH10,MYO1A |
| Sleep NREM Signaling Pathway | 0.008 | -0.707 | ADCY10,ADCY2,GABRA5,GABRD,GABRR2,LOC102724428/SIK1,MRAS,SFN |
| PIP3 activates AKT signaling | 0.009 | -1 | ERBB2,ESR1,FGF9,FGFR4,MYD88,NR4A1,PDGFRB,PPP2R5A,RICTOR |
| Pathogen Induced Cytokine Storm Signaling Pathway | 0.012 | 2.324 | CCL22,COL16A1,COL18A1,COL4A2,CSF2RB,CXCL16,CXCR1,CXCR2,EDA,FTH1,IL1B,IL1RN,MYD88,TGFB2,TLR2 |
| Mitochondrial L-carnitine Shuttle Pathway | 0.013 |  | ACSL1,ACSL6,CPT1B |
| Thioredoxin Pathway | 0.015 |  | TXNDC2,TXNRD3 |
| Glycoaminoglycan-protein Linkage Region Biosynthesis | 0.015 |  | B3GAT1,B4GALT7 |
| RHO GTPases Activate ROCKs | 0.015 |  | LIMK2,MYH10,RHOB |
| PI3K/AKT Signaling | 0.019 | -1 | CSF2RB,CXCR1,CXCR2,IL3RA,ITGA7,ITGA9,ITGAV,MRAS,PPP2R2C,PPP2R5A,SFN |
| Osteoarthritis Pathway | 0.022 | 1.134 | CASQ1,CXCR2,FZD5,IL1B,ITGA7,ITGA9,ITGAV,PTCH1,S1PR3,SMAD1,TLR2 |
| Hepatic Cholestasis | 0.024 | 0.632 | ABCB1,ADCY10,ADCY2,EDA,ESR1,FGFR4,IL1B,IL1RN,MYD88,TGFB2 |
| eNOS Signaling | 0.025 | -0.378 | ADCY10,ADCY2,CCNA1,ESR1,GNAQ,HSPA1A/HSPA1B,HSPA6,LPAR2 |
| Activin Inhibin Signaling Pathway | 0.026 | 1.897 | CCNA1,COL18A1,IL1B,IL1RN,INHBA,LIMK2,MYD88,SMAD1,TGFB2,TLR2 |
| Elastic fibre formation | 0.028 | -1 | FBLN1,ITGAV,LOXL2,TGFB2 |
| HIPPO signaling | 0.030 | -1 | DLG5,PPP2R2C,PPP2R5A,SFN,SMAD1,WWTR1 |
| CDK5 Signaling | 0.030 | -1.134 | ADCY10,ADCY2,LAMC1,MRAS,NTRK2,PPP2R2C,PPP2R5A |
| Granulocyte Adhesion and Diapedesis | 0.031 |  | CCL22,CCR9,CLDN9,CXCL16,CXCR1,CXCR2,IL1B,IL1RN |
| SIRT1 negatively regulates rRNA expression | 0.032 |  | H2BC17,H2BC21,TAF1A |
| Gαs Signaling | 0.033 | 0.816 | ADCY10,ADCY2,ADD2,GNAQ,HCAR2,HCAR3,MRAS |
| PAK Signaling | 0.035 | -1 | ITGA7,ITGA9,ITGAV,LIMK2,MRAS,PAK6,PDGFRB |
| Natural Killer Cell Signaling | 0.035 | 1 | COL18A1,FCGR2A,HSPA1A/HSPA1B,HSPA6,KIR3DL2,KLRC2,LIMK2,MRAS,MYD88,PAK6 |
| Sphingosine-1-phosphate Signaling | 0.039 | 1.134 | ADCY10,ADCY2,CASQ1,GNAQ,PDGFRB,RHOB,S1PR3 |
| Neutrophil degranulation | 0.039 | 4.025 | ATP8B4,CPPED1,CXCR1,CXCR2,FCGR2A,FTH1,HPSE,HSPA1A/HSPA1B,HSPA6,ITGAV,LRG1,MOSPD2,PLAU,RNASET2,SERPINA1,SIGLEC5,SIRPA,TLR2,TNFAIP6,VNN1 |
| Carnitine metabolism | 0.044 |  | ACACB,CPT1B |
| Dissolution of Fibrin Clot | 0.044 |  | PLAU,SERPINE2 |
| L1CAM interactions | 0.044 | -1.134 | ITGA9,ITGAV,KIF4A,LAMC1,NCAM1,NRCAM,TUBB3 |
| Cardiac Hypertrophy Signaling (Enhanced) | 0.045 | 0.5 | ADCY10,ADCY2,ADRA1B,BORCS8-MEF2B,CSF2RB,CXCR1,CXCR2,EDA,FGF9,FGFR4,FZD5,GNAQ,IL1B,IL3RA,ITGA7,ITGA9,ITGAV,MRAS,PDE7B,TGFB2 |
| Ferroptosis Signaling Pathway | 0.046 | -0.378 | CBS/LOC102724560,FTH1,GLS2,H2BC17,H2BC8,MRAS,WWTR1 |
| Transcriptional regulation by the AP-2 (TFAP2) family of transcription factors | 0.047 |  | CITED4,ERBB2,ESR1 |
| Dopamine Receptor Signaling | 0.047 |  | ADCY10,ADCY2,NCS1,PPP2R2C,PPP2R5A |
| GABA Receptor Signaling | 0.049 |  | ADCY10,ADCY2,GABRA5,GABRD,GABRR2,GNAQ,MRAS |

## Table S8. 60 pathways females HR vs females LR (p-value < 0.05)

| **Ingenuity Canonical Pathways** | **p-value** | **z-score** | **Molecules** |
| --- | --- | --- | --- |
| STAT3 Pathway | <0.001 | 1.134 | BMPR1A,CDKN1A,CISH,FGFR2,IGF1,IL11RA,IL12RB2,IL18RAP,IL1RL1,IL4R,IL5RA,MAP3K20,SOCS3 |
| Complement cascade | <0.001 | -1.134 | C3,C4A/C4B,CD55,CFD,CLU,CR1,VTN |
| Inflammasome pathway | <0.001 | -2.236 | AIM2,CASP1,CASP5,NLRC4,NLRP3 |
| Extracellular matrix organization | <0.001 |  | COL4A1,ITGA2B,ITGA3,ITGA8,ITGB4,NID1,NID2,SPARC,TNR,VTN |
| Pathogen Induced Cytokine Storm Signaling Pathway | 0.001 | -1.147 | AIM2,C3,CASP1,CCL23,CCL3L1,COL4A1,CXCL16,CXCL6,HLA-DRB5,IL12RB2,IL18RAP,IL1RL1,IL36A,NLRC4,NLRP3,OSM,SLC2A1,SLC2A3,SOCS3 |
| Osteoarthritis Pathway | 0.001 | -0.302 | ALPL,ANKH,BMPR1A,CASP1,CASP4,CASP5,DDIT4,FZD7,IL18RAP,IL1RL1,ITGA2B,ITGA3,ITGA8,ITGB4,ITGB8 |
| Immunoregulatory interactions between a Lymphoid and a non-Lymphoid cell | 0.001 | -1.508 | C3,CD200R1,FCGR3A/FCGR3B,ITGA3,KIR2DL1/KIR2DL3,KIR2DL4,LILRA5,LILRB2,LILRB4,TREM1,TREML1 |
| Neurotransmitter clearance | 0.001 |  | ACHE,LRTOMT,SLC6A4 |
| Metabolism of porphyrins | 0.002 | -1 | ABCC1,ALAS2,BLVRA,FECH |
| Formation of Fibrin Clot (Clotting Cascade) | 0.002 | -2.236 | CD177,F13A1,GP1BB,PF4V1,SERPINC1 |
| Complement System | 0.002 | 0.447 | C3,C4A/C4B,CD55,CFD,CR1 |
| Integrin cell surface interactions | 0.002 | 1.414 | BSG,COL4A1,FBN1,ITGA2B,ITGA3,ITGA8,ITGB8,VTN |
| DNA damage-induced 14-3-3σ Signaling | 0.002 | -0.816 | BCL2L1,CASP1,CASP4,CASP5,CDKN1A,TP73 |
| PI3K/AKT Signaling | 0.002 | 1 | BCL2L1,CCND1,CDKN1A,IL11RA,IL12RB2,IL18RAP,IL1RL1,IL4R,IL5RA,ITGA2B,ITGA3,ITGA8,ITGB4,ITGB8 |
| Granulocyte Adhesion and Diapedesis | 0.002 |  | CCL23,CCL3L1,CCR9,CXCL16,CXCL6,HRH4,IL18RAP,IL1RL1,IL36A,SDC1,SDC2 |
| IL-10 Signaling | 0.003 | -1.508 | BCL2L1,BLVRA,CCND1,CDKN1A,DDIT4,HLA-DRB5,IL18RAP,IL1RL1,IL36A,IL4R,SOCS3 |
| Cell surface interactions at the vascular wall | 0.004 |  | BSG,CAV1,CD177,FCER1G,IGLL1/IGLL5,ITGA3,PF4V1,SDC1,SDC2,TREM1 |
| Iron homeostasis signaling pathway | 0.005 |  | ABCB10,ALAS2,BMPR1A,CDC34,FECH,HBD,HBG1,HBQ1,HFE,SLC25A37 |
| Class A/1 (Rhodopsin-like receptors) | 0.005 | 1.807 | ADORA3,C3,CCL23,CCL3L1,CCR9,CXCL16,CXCL6,CYSLTR2,HRH4,KEL,NPB,P2RY2,PROK2,PTGDR2,UTS2 |
| Cardiac Hypertrophy Signaling (Enhanced) | 0.005 | 0.943 | ACE,ADCY10,ATP2A1,CACNA1E,CACNB4,CACNG8,FGFR2,FZD7,IGF1,IL11RA,IL12RB2,IL18RAP,IL1RL1,IL36A,IL4R,IL5RA,ITGA2B,ITGA3,ITGA8,ITGB4,ITGB8,MAP3K20,OSM,PDK1,PIK3R6 |
| Caveolar-mediated Endocytosis Signaling | 0.006 |  | CAV1,CD55,ITGA2B,ITGA3,ITGA8,ITGB4,ITGB8 |
| NLR signaling pathways | 0.007 | -2.449 | AIM2,BCL2L1,CASP1,CASP4,NLRC4,NLRP3 |
| IL-13 Signaling Pathway | 0.008 | 0.378 | ALOX15,ALOX15B,BCL2L1,CXCL6,IL4R,PIK3R6,SOCS3 |
| Syndecan interactions | 0.009 | 1 | ITGB4,SDC1,SDC2,VTN |
| Interleukin-4 and Interleukin-13 signaling | 0.011 | -1.414 | ALOX15,BCL2L1,CCND1,CDKN1A,F13A1,IL4R,OSM,SOCS3 |
| Phagosome Formation | 0.014 | 0.784 | ADORA3,C3,CCR9,CLEC4D,CR1,CYSLTR2,FCER1G,FCGR3A/FCGR3B,FZD7,GIPR,GPR146,HRH4,ITGA2B,ITGA3,ITGA8,ITGB4,ITGB8,MARCO,MYL9,MYO10,P2RY2,PIK3R6,PLAAT5,PRDX6,PTGDR2,VTN |
| Role of JAK family kinases in IL-6-type Cytokine Signaling | 0.014 |  | BCL2L1,CCND1,IL11RA,IL27,OSM,SOCS3 |
| PTEN Signaling | 0.014 | -1.633 | BCL2L1,BMPR1A,CCND1,CDKN1A,FGFR2,ITGA2B,ITGA3,ITGA8,ITGB4,ITGB8 |
| LXR/RXR Activation | 0.016 | -0.378 | C3,C4A/C4B,CLU,IL18RAP,IL1RL1,IL36A,VTN |
| TREM1 Signaling | 0.016 | -1.633 | CASP1,CASP5,IL1RL1,NLRC4,NLRP3,TREM1 |
| Amyotrophic Lateral Sclerosis Signaling | 0.016 | -0.447 | BCL2L1,CACNA1E,CACNB4,CACNG8,CASP1,GRINA,IGF1,PIK3R6 |
| Erythrocytes take up oxygen and release carbon dioxide | 0.017 |  | CA1,SLC4A1 |
| The citric acid (TCA) cycle and respiratory electron transport | 0.020 | -0.447 | BSG,HAGH,L2HGDH,ME1,PDK1 |
| FAK Signaling | 0.021 | 1.8 | ADORA3,AGO2,CCND1,CCR9,CYSLTR2,FCER1G,FZD7,GIPR,GPR146,HRH4,IL11RA,IL12RB2,IL18RAP,IL1RL1,IL4R,IL5RA,ITGA2B,ITGA3,ITGA8,ITGB4,ITGB8,P2RY2,PIK3R6,PTGDR2,SOCS3 |
| ID1 Signaling Pathway | 0.022 | 0.905 | BCL2L1,BHLHA15,BMPR1A,CAV1,CCND1,CDKN1A,FGFR2,GSPT1,PIK3R6,RAP1GAP,TGM2 |
| Glycine Betaine Degradation | 0.023 |  | PIPOX,SDSL |
| IL-27 Signaling Pathway | 0.023 |  | CASP1,IL12RB2,IL18RAP,IL1RL1,IL27,NLRP3,PIK3R6,SOCS3 |
| Type II Diabetes Mellitus Signaling | 0.024 |  | ACSF2,ACSM1,ADIPOR1,CACNA1E,CACNB4,CACNG8,PIK3R6,SMPD3,SOCS3 |
| IL-4 Signaling | 0.025 | -0.632 | ALOX15,ALOX15B,BCL2L1,COL4A1,FCER1G,HLA-DRB5,IL4R,PIK3R6,PTGDR2,TGM2 |
| Retinoate Biosynthesis I | 0.025 |  | AKR1C3,ALDH1A2,HSD17B6 |
| Arachidonic acid metabolism | 0.025 | 1 | ABCC1,ALOX15,ALOX15B,FAAH |
| Th2 Pathway | 0.026 |  | HLA-DRB5,IL12RB2,IL1RL1,IL4R,NOTCH4,PIK3R6,PTGDR2,SOCS3 |
| Interleukin-6 family signaling | 0.028 |  | IL11RA,OSM,SOCS3 |
| Heme Biosynthesis II | 0.029 |  | ALAS2,FECH |
| Pyroptosis Signaling Pathway | 0.030 | -2.449 | AIM2,CASP1,CASP4,CASP5,NLRC4,NLRP3 |
| Glycosaminoglycan metabolism | 0.030 |  | B4GALT5,B4GALT7,CHPF,SDC1,SDC2,ST3GAL4 |
| Elastic fibre formation | 0.030 | 2 | FBN1,ITGA8,ITGB8,VTN |
| Graft-versus-Host Disease Signaling | 0.030 |  | FCER1G,HLA-DRB5,IL36A,KIR2DL1/KIR2DL3 |
| Histamine Biosynthesis | 0.030 |  | HDC |
| Regulation of Cellular Mechanics by Calpain Protease | 0.035 |  | CCND1,ITGA2B,ITGA3,ITGA8,ITGB4,ITGB8 |
| Androgen Biosynthesis | 0.035 |  | AKR1C3,HSD17B6 |
| Th1 and Th2 Activation Pathway | 0.037 |  | HLA-DRB5,IL12RB2,IL1RL1,IL27,IL4R,NOTCH4,PIK3R6,PTGDR2,SOCS3 |
| G alpha (i) signalling events | 0.040 | 2.111 | ADORA3,C3,CCL23,CCR9,CXCL16,CXCL6,HRH4,NPB,PTGDR2,RGS6,TAS2R14 |
| HGF Signaling | 0.042 | 2 | CCND1,CDKN1A,ITGA2B,ITGA3,ITGA8,ITGB4,ITGB8,PIK3R6 |
| Erythrocytes take up carbon dioxide and release oxygen | 0.043 |  | CA1,SLC4A1 |
| Extrinsic Prothrombin Activation Pathway | 0.043 |  | F13A1,SERPINC1 |
| Pyroptosis | 0.044 |  | CASP1,CASP4,CASP5 |
| CGAS-STING Signaling Pathway | 0.047 | -0.378 | ABCC1,CASP1,CDKN1A,IDO1,IL36A,NLRP3,OSM |
| LPS/IL-1 Mediated Inhibition of RXR Function | 0.048 |  | ABCB9,ACSF2,ACSM1,ALDH1A2,CHST10,CRAT,GSTM2,IL18RAP,IL1RL1,IL36A |

## Table S9. 26 pathways males MDD vs males HR (p-value < 0.05)

| **Ingenuity Canonical Pathways** | **p-value** | **z-score** | **Molecules** |
| --- | --- | --- | --- |
| Mitotic Roles of Polo-Like Kinase | <0.001 | 1.342 | CDC20,CDC25A,ESPL1,KIF11,PKMYT1,PLK1,PPM1J |
| Extracellular matrix organization | <0.001 | 2.121 | COL1A2,COL4A4,COL5A3,DCN,ITGA8,MATN1,PDGFB,SERPINE1 |
| Cell Cycle Checkpoints | 0.002 | 3.606 | AURKB,BUB1,CDC20,CDC25A,CENPF,CLSPN,ERCC6L,GTSE1,KNL1,ORC1,PKMYT1,PLK1,PSMB11 |
| Kinetochore Metaphase Signaling Pathway | 0.003 | 0.378 | AURKB,BUB1,CDC20,ESPL1,KNL1,PLK1,SKA3 |
| Choline catabolism | 0.003 |  | ALDH7A1,SLC44A1 |
| RHO GTPases Activate Formins | 0.004 | 2.828 | AURKB,BUB1,CDC20,CENPF,DAAM1,ERCC6L,KNL1,PLK1 |
| Intrinsic Prothrombin Activation Pathway | 0.009 |  | COL1A2,COL5A3,KLK1 |
| Syndecan interactions | 0.013 |  | COL1A2,COL5A3,SDC1 |
| Asparagine Biosynthesis I | 0.019 |  | ASNS |
| Neurexins and neuroligins | 0.019 |  | EPB41L1,LIN7B,LRRTM3,NLGN4Y |
| Kinesins | 0.019 | 2 | KIF11,KIF18B,KIF4A,KIFC1 |
| Dissolution of Fibrin Clot | 0.020 |  | PLG,SERPINE1 |
| Signaling by PDGF | 0.020 | 1 | COL4A4,COL5A3,PDGFB,PLG |
| Role of CHK Proteins in Cell Cycle Checkpoint Control | 0.023 |  | CDC25A,CLSPN,PLK1,PPM1J |
| Immunoregulatory interactions between a Lymphoid and a non-Lymphoid cell | 0.023 | -1.633 | CD34,COL1A2,COLEC12,CRTAM,LILRB1,SFTPD |
| Collagen biosynthesis and modifying enzymes | 0.027 | 2 | ADAMTS2,COL1A2,COL4A4,COL5A3 |
| Mitotic Metaphase and Anaphase | 0.030 | 3 | AURKB,BUB1,CDC20,CENPF,ERCC6L,ESPL1,KNL1,PLK1,PSMB11 |
| Adenosine Nucleotides Degradation II | 0.031 |  | ADARB2,NT5C3B |
| Collagen chain trimerization | 0.035 |  | COL1A2,COL4A4,COL5A3 |
| Alanine metabolism | 0.037 |  | GPT2 |
| Choline Degradation I | 0.037 |  | ALDH7A1 |
| Alanine Degradation III | 0.037 |  | GPT2 |
| Alanine Biosynthesis II | 0.037 |  | GPT2 |
| Atherosclerosis Signaling | 0.038 |  | COL1A2,COL5A3,LPL,PDGFB,PLA2G2D |
| Transport of bile salts and organic acids, metal ions and amine compounds | 0.040 | 1 | SLC13A4,SLC44A1,SLC5A11,SLC6A20 |
| Purine Nucleotides Degradation II (Aerobic) | 0.044 |  | ADARB2,NT5C3B |

## Table S10. 50 pathways males MDD vs males LR (p-value < 0.05)

| **Ingenuity Canonical Pathways** | **p-value** | **z-score** | **Molecules** |
| --- | --- | --- | --- |
| Mitotic Roles of Polo-Like Kinase | <0.001 | 1.89 | CCNB1,CDC20,CDC25A,CDK1,ESPL1,HSP90B1,KIF11,PKMYT1,PLK1,PPP2R3A |
| Estrogen-mediated S-phase Entry | <0.001 | 2.449 | CCNA2,CDC25A,CDK1,E2F2,E2F7,E2F8 |
| Kinetochore Metaphase Signaling Pathway | <0.001 | 1.508 | AURKB,BUB1B,CCNB1,CDC20,CDK1,CENPE,ESPL1,KNL1,MAD1L1,PLK1,ZWINT |
| Cell Cycle Checkpoints | <0.001 | 3.771 | AURKB,BUB1B,CCNA2,CCNB1,CDC20,CDC25A,CDC6,CDK1,CENPE,CENPM,CLSPN,GTSE1,KNL1,MAD1L1,PKMYT1,PLK1,UBE2C,ZWINT |
| Role of CHK Proteins in Cell Cycle Checkpoint Control | <0.001 | -2.449 | CDC25A,CDK1,CLSPN,E2F2,E2F7,E2F8,PLK1,PPP2R3A |
| RHO GTPases Activate Formins | <0.001 | 2.309 | AURKB,BUB1B,CDC20,CENPE,CENPM,DAAM1,KNL1,MAD1L1,PLK1,RHOD,TUBB2A,ZWINT |
| Kinesins | <0.001 | 1.134 | CENPE,KIF11,KIF26B,KIF4A,KIF5A,KIFC1,TUBB2A |
| Cyclins and Cell Cycle Regulation | 0.001 | 2.828 | CCNA2,CCNB1,CDC25A,CDK1,E2F2,E2F7,E2F8,PPP2R3A |
| TP53 Regulates Transcription of Cell Cycle Genes | 0.001 | 2.449 | CCNA2,CCNB1,CDK1,E2F7,E2F8,PLAGL1 |
| Regulation of mitotic cell cycle | 0.001 | 2.828 | AURKB,BUB1B,CCNA2,CCNB1,CDC20,CDK1,PLK1,UBE2C |
| Mitotic Metaphase and Anaphase | 0.001 | 2.673 | AURKB,BUB1B,CCNB1,CDC20,CDK1,CENPE,CENPM,ESPL1,KNL1,MAD1L1,PLK1,TUBB2A,UBE2C,ZWINT |
| Neutrophil degranulation | 0.001 | 3.838 | ABCA13,AZU1,CEACAM6,CEACAM8,CHIT1,CLEC4C,CRISP3,DEFA1 (includes others),DEFA4,ELANE,FOLR3,GGH,HP,LCN2,LTF,MMP8,MPO,OLFM4,RETN,RNASE2,SLC2A5,TXNDC5 |
| HEY1 Signaling Pathway | 0.001 | 2.53 | BMP6,BMP8A,BMP8B,E2F2,E2F7,E2F8,JAG1,MMP8,NOTCH3,VEGFC |
| Mitotic Prometaphase | 0.002 | 2.309 | AURKB,BUB1B,CCNB1,CDC20,CDK1,CENPE,CENPM,KNL1,MAD1L1,PLK1,TUBB2A,ZWINT |
| Epithelial Adherens Junction Signaling | 0.002 | -1.265 | AFDN,CTNNA2,MAGI1,MAGI2,MET,MYH10,NECTIN2,NOTCH3,PPP2R3A,WASF1 |
| Threonine catabolism | 0.003 |  | GCAT,SDS |
| Extracellular matrix organization | 0.005 | 1.134 | CEACAM6,CEACAM8,COL4A4,DCN,ITGA8,PTPRS,TNR |
| Binding and Uptake of Ligands by Scavenger Receptors | 0.007 | 1 | COLEC12,HP,HSP90B1,JCHAIN |
| Phospholipases | 0.007 |  | GPLD1,LPL,PLA2G12A,PLAAT2,PNPLA4 |
| Cell Cycle Regulation by BTG Family Proteins | 0.011 |  | E2F2,E2F7,E2F8,PPP2R3A |
| Glutathione-mediated Detoxification | 0.013 |  | GGH,GSTA1,PTGES |
| Butyrophilin (BTN) family interactions | 0.013 |  | BTN1A1,CD209 |
| Osteoarthritis Pathway | 0.016 | 0.378 | CTNNA2,DCN,ELF3,FZD7,IL18RAP,ITGA8,JAG1,PPARG,PPARGC1A,VEGFC |
| Defensins | 0.017 |  | DEFA1 (includes others),DEFA4 |
| Sphingosine and Sphingosine-1-phosphate Metabolism | 0.017 |  | ASAH2B,SGPP2 |
| FOXO-mediated transcription of oxidative stress, metabolic and neuronal genes | 0.017 |  | ABCA6,PPARGC1A,RETN |
| Pulmonary Healing Signaling Pathway | 0.020 | 1 | CCNB1,CTRC,ELANE,FZD7,JAG1,MMP8,NOTCH3,PRKD1,VEGFC |
| Cell Cycle: G1/S Checkpoint Regulation | 0.020 | -2.236 | CDC25A,E2F2,E2F7,E2F8,NRG1 |
| Cell surface interactions at the vascular wall | 0.023 | 1.134 | CEACAM6,CEACAM8,IGLL1/IGLL5,JCHAIN,SDC1,SDC2,SLC7A9 |
| Melatonin Degradation III | 0.023 |  | MPO |
| Glutamate Removal from Folates | 0.023 |  | GGH |
| Threonine Degradation II | 0.023 |  | GCAT |
| Cell Cycle: G2/M DNA Damage Checkpoint Regulation | 0.030 | -1 | CCNB1,CDK1,PKMYT1,PLK1 |
| Retinoid metabolism and transport | 0.032 |  | LPL,SDC1,SDC2 |
| Inhibition of Matrix Metalloproteases | 0.032 |  | MMP8,SDC1,SDC2 |
| Glycosaminoglycan metabolism | 0.032 | 1.342 | B4GALT6,CHPF,DCN,SDC1,SDC2 |
| Folate Signaling Pathway | 0.034 |  | ADARB2,CCNA2,FOLR3,GGH |
| Regulation of TP53 Activity through Association with Co-factors | 0.035 |  | TP63,TP73 |
| Azathioprine ADME | 0.035 |  | GSTA1,SLC28A3 |
| Crosstalk between Dendritic Cells and Natural Killer Cells | 0.035 |  | CD209,CD80,FSCN2,NECTIN2,TLN2 |
| Mitotic G2-G2/M phases | 0.037 | 2.333 | CCNA2,CCNB1,CDC25A,CDK1,GTSE1,PKMYT1,PLK1,TPX2,TUBB2A |
| Role of BRCA1 in DNA Damage Response | 0.037 | -0.447 | E2F2,E2F7,E2F8,FAAP24,PLK1 |
| Neurexins and neuroligins | 0.038 | -1 | APBA1,EPB41L1,NLGN4Y,NRXN2 |
| Transcriptional regulation of white adipocyte differentiation | 0.039 | 1.342 | KLF5,LPL,PLIN1,PPARG,PPARGC1A |
| Triacylglycerol Degradation | 0.040 |  | FAAH,LPL,PNPLA4 |
| Neutrophil Extracellular Trap Signaling Pathway | 0.041 | 1.387 | CD209,COL4A4,DEFA1 (includes others),DEFA4,ELANE,JCHAIN,KCNN3,LTF,MPO,PLA2G12A,PLAAT2,PNPLA4,PRKD1 |
| Primary Immunodeficiency Signaling | 0.043 |  | IGLL1/IGLL5,JCHAIN,TNFRSF13B |
| Keratinization | 0.045 | 1 | DSC2,DSG2,PERP,PRSS8 |
| Wax and plasmalogen biosynthesis | 0.046 |  | FAR2 |
| Glycerol-3-phosphate Shuttle | 0.046 |  | GPD1 |

## Table S11. 26 pathways males HR vs males LR (p-value < 0.05)

| **Ingenuity Canonical Pathways** | **p-value** | **z-score** | **Molecules** |
| --- | --- | --- | --- |
| Oxidative Phosphorylation | 0.006 | -2.646 | ATP5ME,ATP5PF,ATP5PO,COX17,NDUFA1,NDUFS5,UQCRB |
| Cell junction organization | 0.008 |  | CADM2,CD151,CDH5,CLDN12,NECTIN2,PRKCI |
| Phase II - Conjugation of compounds | 0.011 | 1 | AS3MT,GGT5,SLC26A1,SULT1A3/SULT1A4 |
| Hematopoiesis from Pluripotent Stem Cells | 0.011 |  | CXCL8,FCER1G,IL12A |
| Transcriptional regulation of testis differentiation | 0.012 |  | AMH,PTGDS |
| Role of Cytokines in Mediating Communication between Immune Cells | 0.013 |  | CXCL8,IL12A,IL15 |
| Nonsense-Mediated Decay (NMD) | 0.013 | -2.646 | RBM8A,RPL22,RPL26,RPL36A,RPL36AL,RPS23,UPF3B |
| Transport of inorganic cations/anions and amino acids/oligopeptides | 0.013 | 2.449 | SLC12A1,SLC25A29,SLC26A1,SLC4A10,SLC4A4,SLC4A7 |
| Metabolism of steroid hormones | 0.015 |  | CYP21A2,HSD17B14,STARD4 |
| Granzyme A Signaling | 0.018 | 0.447 | CASP1,H1-2,MT-ND6,NDUFA1,NDUFS5 |
| Chondroitin Sulfate Biosynthesis | 0.019 |  | B3GAT2,HS3ST1,SULT1A3/SULT1A4,UST |
| Acetylcholine binding and downstream events | 0.019 |  | CHRNA4,CHRNA5 |
| Electron transport, ATP synthesis, and heat production by uncoupling proteins | 0.021 | -1.89 | ATP5ME,ATP5PF,ATP5PO,MT-ND6,NDUFA1,NDUFS5,UQCRB |
| Dermatan Sulfate Biosynthesis | 0.023 |  | B3GAT2,HS3ST1,SULT1A3/SULT1A4,UST |
| Cristae formation | 0.029 |  | ATP5ME,ATP5PF,ATP5PO |
| Metabolism of Angiotensinogen to Angiotensins | 0.032 |  | AGT,CPA3 |
| Methylglyoxal Degradation III | 0.032 |  | PTGR1,PTGR2 |
| Keratinization | 0.035 | -1 | KRT72,KRT73,KRT8,PPL |
| Nicotinate metabolism | 0.036 |  | NMRK1,RNLS |
| Synthesis of Prostaglandins (PG) and Thromboxanes (TX) | 0.036 |  | PTGDS,PTGR2 |
| SRP-dependent cotranslational protein targeting to membrane | 0.036 | -2.449 | RPL22,RPL26,RPL36A,RPL36AL,RPS23,SRP14 |
| Heparan Sulfate Biosynthesis | 0.041 |  | B3GAT2,HS3ST1,SULT1A3/SULT1A4,UST |
| Choline Degradation I | 0.043 |  | CHDH |
| Dermatan Sulfate Biosynthesis (Late Stages) | 0.046 |  | HS3ST1,SULT1A3/SULT1A4,UST |
| Communication between Innate and Adaptive Immune Cells | 0.048 |  | CXCL8,FCER1G,IL12A,IL15 |

*Table S11. Table of the numerosity of the genes differentially expressed before and after the filter applied for the analysis.*

|  | **MDD vs HR** | | | **HR vs LR** | | | **MDD vs LR** | | |
| --- | --- | --- | --- | --- | --- | --- | --- | --- | --- |
| **Transcript counts** | **ALL** | **Female** | **Male** | **ALL** | **Female** | **Male** | **ALL** | **Female** | **Male** |
| Total | 60230 | 60230 | 60230 | 60230 | 60230 | 60230 | 60230 | 60230 | 60230 |
| After filtering rows <10 reads | 30462 | 27630 | 27940 | 30321 | 27630 | 27940 | 30383 | 27485 | 27946 |
| After filtering NA genes | 21356 | 20092 | 20235 | 21296 | 20092 | 20235 | 21339 | 20019 | 20237 |
| After filtering non protein-coding transcripts | 16802 | 16252 | 16337 | 16786 | 16252 | 16337 | 16774 | 16249 | 16334 |
