# Supplementary material for: Inflammation and immune system pathways as biological signatures of adolescent depression—the IDEA-RiSCo study
Source: Transl Psychiatry. 2024 Jun 1;14:230. doi: 10.1038/s41398-024-02959-z (PMC11144232; doi:10.1038/s41398-024-02959-z)
Supplement: Supplementary file 4 — Supplementary Methods [file 41398_2024_2959_MOESM4_ESM.docx]

##

## SUPPLEMENTARY MATERIAL

## IDEA (Identify Depression Early in Adolescence) Project sample recruitment

### Ethical approval

The study was approved by the Brazilian National Ethics Committee, project number 50473015.9.0000.5327, the Hospital de Clinicas de Porto Alegre’s Ethics Committee, project number 16-0131 and the King’s College’s Institutional Research Board, project number LRS-17/18-8327.

Adolescents and their primary caregivers provided written consent prior to entering the study. Approval for the school screening phase was obtained from the 1st Regional Education Bureau, in charge of public state schools in the city of Porto Alegre. All participants received feedback with findings from the diagnostic assessment and were referred for care to the Brazilian public health system if clinically indicated. Situations of imminent risk of self-harm or maltreatment were referred to emergency care or protective services following what required by Brazilian legislation. Participants received no financial incentive for taking part in the study, however they were reimbursed for expenses related to their participation (e.g., travel).

The recruitment of participants was conducted in 4 stages (Table 3.1): school screening, telephone interview, clinical assessment, and biological sample collection (Kieling et al., 2021).

###### Criteria for the IDEA Risk Stratified Cohort sample composition

| **Phase** | **Inclusion criteria** | **Exclusion criteria** |
| --- | --- | --- |
| *School screening* | Enrolment in 8^th^ to 11^th^ grades  Age 14 to 16 years  Right-handedness | Absent from school on the day of both assessments  Inability to complete the screening questionnaire |
| *Phone invitation* | Completed school questionnaire | Metallic accessories  Clinical conditions*  Use of psychotropic medication over last 30 days  Use of anti-inflammatory medication over last 14 days |
| *Clinical interview* | IQ > 70  Post-pubertal status | Current or lifetime:  Bipolar disorder  Schizophrenia or a primary psychotic disorder  Autism spectrum disorder  Substance use disorder  Eating disorder  Post-traumatic stress disorder |

**Excluded clinical conditions: known brain malformations, epilepsy, recent traumatic brain injury, diabetes, cystic fibrosis, HIV, asthma, rheumatologic conditions such as rheumatoid arthritis, systemic lupus erythematosus, psoriasis, purpura, oncologic conditions such as cancer, lymphoma, leukemia, and severe neurodevelopmental disorders, any recent/active infection or active inflammatory process.*

### School screening

For the school screening the only inclusion criterion was being aged 14 to 16 years old. The Brazilian recruiting team, led by Dr Christian Kieling, first contacted the Department of Education from Porto Alegre to get permission to contact school representatives. Then, they arranged with schools for eligible students to take part to the recruitment.

The recruiting team used a screening questionnaire, which was divided in two sequential parts: an open questionnaire with a brief identification form and questions on social media usage and physical activity; and a confidential questionnaire comprised of the Patient Health Questionnaire for Adolescents (PHQ-A), questions on drug use and fight involvement, parental relationship and seven dichotomous questions on lifetime sexual, emotional, or physical maltreatment experiences.

The PHQ-A is an adapted version from the PHQ-9 to be specifically used with adolescents as a screening tool in both clinical (Johnson, Harris, Spitzer, & Williams, 2002) and research settings (Allgaier et al., 2012). It consists of 9 questions with Likert-type response options ranging from “none”, “several days” and “more than half the days”. Each of the 9 items are designed to represent the nine DSM-5 criteria for a Major Depressive episode. The scale was translated from English to Brazilian Portuguese by the research team.

### Risk Score

The composite IDEA risk score (IDEA-RS) developed by the IDEA consortium was used for the risk-stratification of the adolescent sample (Rocha et al., 2021). This risk score does not consider previous depressive symptomatology as a risk factor for MDD, but information that could be easily obtained from the adolescents to increase its feasibility use in wider scenarios. The risk score was built up by using a short questionnaire that comprises the following variables:

- Biological Sex
- Skin colour
- Drug use
- School failure
- Social Isolation
- Fight Involvement
- Poor relationship with the mother
- Poor relationship with the father
- Poor relationship between parents
- Childhood Maltreatment
- Ran away from home

The lifetime maltreatment experiences were divided into three categories, accordingly to the previous study of Rocha and colleagues (Rocha, Graeff-Martins, Kieling, & Rohde, 2015): no maltreatment (no positive answer), probable maltreatment (one positive answer), and severe maltreatment (two or more positive answers). The predictive risk score presented different percentile cut-offs for males and females, due to the discrepancy in prevalence between sexes.

Administration of the IDEA-RS questionnaire in the schools was performed using a coded, unidentified form distributed to students after information on name, date of birth, self-reported sex, self-reported race/skin colour, handedness, and parental contact information were collected. Questions were selected to match the original phrasing used in the Pelotas 1993 Birth Cohort study. On average, less than 15 minutes were required for administration of the IDEA-RS questionnaire. Students were allowed to ask clarification questions, but researchers were not allowed to review the form to check for completion (forms were considered to be “complete” and therefore valid when only one answer was provided for each question and all questions were answered). The questionnaire is reported in Table 3.2 (Kieling et al., 2021).

###### Table 3.2. The Identifying Depression Early in Adolescence Risk Score (IDEA-RS). From (Kieling et al., 2021).

** Self-reported skin colour following Brazilian official census categories. For analyses, two categories (white vs. non-white) were formed.*

*** Questions about any lifetime use of alcohol, tobacco, cannabis, cocaine, and inhalants were combined into one variable using the OR rule, generating a binary variable for analyses.*

**** Responses to seven dichotomous questions regarding lifetime psychological, physical, and sexual abuse and/or neglect were combined into three categories: zero positive answers=none, 1 positive=probable, 2 or more answers=severe.*

| Sex: | Male/Female |
| --- | --- |
| Your skin colour or race is:* | White/Yellow/Indigenous/  Brown/Black |
| Do you meet your friends often to talk, play or do anything else? | No/yes |
| Have you ever failed a school grade? | No/yes |
| Have you ever run away from home? | No/yes |
| Have you ever tried cigarettes?** | No/yes |
| Have you ever tried alcohol?** | No/yes |
| Have you ever tried sniffing glue?** | No/yes |
| Have you ever tried sniffing solvents or ethyl chloride (EC)?** | No/yes |
| Have you ever tried marijuana?** | No/yes |
| Have you ever tried cocaine or crack?** | No/yes |
| Have you ever tried LSD or acid?** | No/yes |
| Have you ever tried ecstasy or molly?** | No/yes |
| Have you ever used weight loss pills?** | No/yes |
| Have you ever used tranquilizers or sleeping pills?** | No/yes |
| Have you ever used any drug? ** | No/yes |
| In the last year, did you get into any fight in which somebody got hurt? | No/yes |
| Would you say your relationship with your father is: | Great/Very good/  Good/Regular/Bad |
| Would you say your relationship with your mother is: | Great/Very good/  Good/Regular/Bad |
| Would you say the relationship between your father and mother is: | Great/Very good/  Good/Regular/Bad |
| Have you ever been separated from your parents so that you had to stay with someone else?*** | No/yes |
| At home, have you witnessed fights with physical aggression between adults, or has any adult assaulted a child or teenager?*** | No/yes |
| Have you experienced not having enough food at home, or have you had to wear dirty or torn clothes because you had no other?*** | No/yes |
| Have you ever thought or felt that your parents wished you were never born?*** | No/yes |
| Have you ever thought or felt that someone in your family hated you?*** | No/yes |
| Have you ever been beaten by an adult in your family or by someone who was taking care hard enough to leave marks or hurt you?*** | No/yes |
| Has anyone ever tried to touch you in a sexual way, or tried to make you touch them against your will, threatening you or hurting you?*** | No/yes |

Using cut-offs for the IDEA-RS based on the Pelotas 1993 Birth Cohort Study (Rocha et al., 2021), eligible participants were *a priori* stratified into two different cohorts:

- Low-risk (LR) adolescents: those scoring equal to or below the 20^th^ percentile of the IDEA-RS
- High-risk (HR) adolescents: those scoring equal to or above the 90^th^ percentile of the IDEA-RS.

A larger stratum in the LR group compared the HR group was allowed as the absolute risk difference between the 10^th^ and the 20^th^ percentiles was minimal. Moreover, a third group of adolescents with MDD was recruited. To allow for two-by-two comparisons between groups, the adolescents in the MDD group were also required to be classified as HR risk, so with a score equal to or above the 90^th^ percentile of the IDEA-RS.

To optimize the recruitment process and increase the probability that diagnostic criteria for depression were met in the MDD group, but not in the LR and HR groups, during the school screening adolescents also completed the Patient Health Questionnaire—adolescent version (PHQ-A) (Johnson et al., 2002). Specifically, adolescents with a PHQ-A ≤ 6 were considered for further assessment for the LR/HR groups, whereas adolescents with a PHQ-A ≥ 10 for the MDD group.

Overall, by combining the risk prediction score and the total PHQ-A score, the participants were divided in three eligible groups for clinical assessment invitation:

1. High risk (HR): PHQ-A score lower than or equal to 6 AND risk score above the 90^th^ percentile
2. Low risk (LR): PHQ-A score lower than or equal to 6 AND risk score below the 20^th^ percentile
3. Depression group (MDD): PHQ-A score higher than or equal to 10, as suggested in the literature and risk score above the 90^th^ percentile of risk

### Telephone interview

After participants were divided in the three eligible groups, the recruiting team contacted them via a telephone interview to assess possible exclusion criteria and schedule the clinical assessment. These exclusion criteria applied were:

- current (i.e., in the last two weeks) use of psychotropics, antibiotics, beta-blockers or anti-inflammatory medication;
- presence of metallic implants (dental braces, piercings, pacemaker, etc.);
- current active infection;
- presence of clinical comorbidities requiring current treatment (HIV, asthma, epilepsy, brain malformations, diabetes, cystic fibrosis, etc.);
- history of cranioencephalic trauma or concussions; pregnancy; and recent (i.e., past 3 months) tattoos.

If adolescents were willing to participate, an appointment was scheduled and adolescents were instructed to be fasting, not smoking, or drinking alcohol, avoiding strenuous physical activity, and not interrupting any continuous non-excludent medication (e.g., oral contraceptives).

### Clinical Assessment

After the school screening, adolescents meeting the criteria for further assessments were evaluated by trained psychologists for cognitive measurements and by skilled child psychiatrists for clinical diagnoses. Before proceeding with the clinical assessment, the child psychiatrist reassessed the telephone interview exclusion criteria, especially for recent use of medications. Absence of a lifetime history of depression for the HR and LR groups was assessed using the Brazilian Portuguese translation of the Schedule for Affective Disorders and Schizophrenia for School-age Children-Present and Lifetime Version (K-SADS-PL) by trained clinicians. Participants in all three groups were excluded if they met lifetime diagnostic criteria for autism spectrum disorder, bipolar disorder, eating disorders, post-traumatic stress disorder, schizophrenia, or substance use disorders.

Youth assigned to LR, HR and MDD group underwent phenotypic assessment; psychological and socio-environmental assessments including self- and clinician-based are detailed below and in Table 3.3 and are listed below (only those relevant for the recruitment are described), for both adolescents and primary caregivers.

- DSM-5 Self-Rated Level 1 Cross-Cutting Symptom Measure—Child Age 11–17

The DSM-5 Cross-Cutting symptom scale is a self-report instrument for the assessment of twelve psychiatric constructs over the last two weeks including depressive symptomatology, anger, irritability, mania, anxiety, somatic symptoms, inattention, suicidal ideation and attempts, psychosis, sleep troubles, repetitive thought and behaviours, and substance use (Bastiaens & Galus, 2018).

- Wechsler Abbreviated Scale of Intelligence (WASI)
- Parental Bonding Instrument (PBI)
- Self-Administered Physical Activity Checklist
- Mood and Feelings Questionnaire (MFQ)
- Childhood Trauma Questionnaire (CTQ)

The CTQ is a self-report measure of traumatic experiences consisting of 28 items that assess 5 dimensions: physical abuse and neglect, emotional abuse and neglect and sexual abuse. It has been translated and validated to Brazilian Portuguese (Grassi-Oliveira et al., 2014).

- Snaith-Hamilton Pleasure Scale (SHAPS)
- Reflective Functioning Questionnaire for Youths (RFQY)
- Affective Reactivity Index (ARI)
- Mood Disorder Questionnaire (MDQ)
- Spence Children's Anxiety Scale (SCAS)
- The Youth Strength Inventory – Adolescent version (YSI-A)
- Adolescent Resilience Scale (ARS)
- U–Change Home Questionnaire Pack
- Tanner Puberty Staging Scale
- Brazilian Economic Classification Criteria (ABEP)
- Kiddie – Schedule for Affective Disorders and Schizophrenia for School-Age Children – Present and Lifetime Version (K-SADS-PL)

The K-SADS-PL is a semi-structured psychiatric diagnostic interview that assesses present and lifetime conditions (Kaufman et al., 1997). K-SADS interviews will be used in this study as the clinician-rated diagnostic criteria for Major Depressive Disorder and other psychiatric conditions.

- Clinical Global Impression (CGI)
- Children Depression Rating Scale (CDRS-R)
- Children’s Global Assessment Scale (CGAS)

### IDEA RiSCo Recruitment – Flowchart and details

Following the steps of the recruitment previously explained, now the details in terms of numbers of adolescents screened and recruited will be described.

In the city of Porto Alegre (Brazil) during the 2018, 104 public state schools agreed to participate to the IDEA project recruitment, for a total of 24,559 students in grades 8 to 11. Of this initial population of 24,559 students, 13,408 students were eligible for screening, whereas 11,151 were excluded because 9,191 were out of age (<14 years or ≥17 years); 1,439 were transferred to another state school; 327 cancelled enrollment; and 194 were excluded for cognitive impairment, language barriers, previous participation in the protocol at another school, or death.

On the day of the parent information form (PIF) distribution, the PIF was distributed to 10,529 students who became eligible to complete the screening; however, only 6,863 students completed the first questionnaire round (Q1). On the other hand, Q2 was administered to 857 students. Considering Q1 and Q2 administration, a total of 7,720 students completed screening questionnaires in 101 schools. The detailed flowchart is reported in figure 3.1, showing the exclusion criteria for each step.

Out of the 7,720 adolescents, 1,766 were eligible for further assessment: 369 were classified as LR, 389 as HR, and 1,008 as MDD. Parents or guardians were contacted over the phone and invited to accompany the adolescent to the Hospital de Clínicas de Porto Alegre (HCPA). Contact with 21, 81, and 506 participants in the LR, HR, and MDD groups respectively was not attempted because the target sample was met before they were called. In the LR group 348 were contacted, among them 78 adolescents were scheduled for clinical evaluation and 64 of them underwent clinical evaluation. In the HR group, 308 were contacted, 97 adolescents were scheduled for clinical evaluation and 63 of them underwent clinical evaluation. In the MDD group, 502 were contacted, 166 adolescents were scheduled for clinical assessment and 133 of them underwent clinical evaluation. Among them, 50 per group (25 males and 25 females) were recruited, and the recruitment stopped once the target sample size of 50 per group was reached. Specifically, participants were stratified into the three groups according to the presence/absence of major depressive disorder and to their level of risk for developing depression based on the IDEA risk score (IDEA-RS), which was previously developed to estimate the future probability of a diagnosis of major depressive disorder in adolescence (Rocha et al., 2021) The three groups included: 1) one group of adolescents at low risk of developing depression (n=50), 2) one group of adolescents at high risk of developing depression (n=50), and 3) one group of adolescents currently meeting diagnostic criteria for major depressive disorder (n=50). Non-depressed low-risk (LR) adolescents were those scoring equal to or below the 20th percentile of the IDEA-RS and ≤ 6 on the Patient Health Questionnaire—Adolescent Version (PHQ-A). Non-depressed high-risk (HR) adolescents were those scoring equal to or above the 90th percentile of the IDEA-RS and ≤ 6 on the PHQ-A. The group with depression scored equal or above 90th percentile of the IDEA-RS and PHQ-A ≥10.

## Pathways analysis and Ingenuity Pathway Analysis Software

Genes differentially modulated across groups were then used to run a pathway analysis by using Ingenuity Pathway Analyses Software (Qiagen). Specifically, IPA software requires to upload lists of genes including columns of measurements about each gene, such as fold-changes and p-values. The software detects the pathway overlap of the significant dataset molecules, by using an algorithm that compares the up-regulated and the down-regulated genes in the uploaded data to the pattern expected for that specific pathway. Indeed, up-regulated genes might activate or inhibit the pathways, depending on the role of that genes which is thus accurately balanced and recognized by the software. The results of the pathway analysis are reported in the “Core Analysis” tab, which reported the lists of pathways significantly associated with the list of genes differently expressed, together with their fold-change and p-values. The statistical significance of the overlap of the uploaded datasets of DEGs is calculated by using the Fisher’s Exact Test. The calculation of the p-value for the identification of the pathways differentially expressed depends on several factor, specifically: 1) the number of molecules associated with a given pathways; 2) the number of eligible analysis-ready molecules from the database that participate in the annotation and are also in the uploaded reference set of genes; 3) the total number of molecules known to be associated with that annotation and that are in the reference set of DEGs uploaded; 4) the total number of molecules in the reference set; 5) the total number of analysis-ready molecules that did not match that annotation. However, the IPA software does not apply a Bonferroni correction or any other

correction because of a possible overcorrection of the results, thus leading to a high

false negative rate accordingly to the IPA user manual. Moreover, as reported in the user manual, it is recommended to not use multiple testing corrections when analysing canonical pathways, and this advice was followed in this paper. Each pathway will be reported with its p-value and its z-score. Conceptually, the z-score is a statistical measure of how closely the actual expression pattern of the DEGs in the uploaded datasets compare to the pattern that is expected based on the literature for each specific pathway identified. The possible results that IPA provides in terms of z-score are: i) z-score>2 meaning a predicted activation of that pathway (an up-regulation), ii) z-score<-2 meaning a predicted inactivation (a down-regulation); iii) no z-score, meaning that the software was not able to identify whether the pathway was activated or inactivated based on the DEGs provided (Kramer, Green, Pollard, & Tugendreich, 2014).

## REFERENCES

Allgaier, A. K., Pietsch, K., Fruhe, B., Prast, E., Sigl-Glockner, J., & Schulte-Korne, G. (2012). Depression in pediatric care: is the WHO-Five Well-Being Index a valid screening instrument for children and adolescents? *Gen Hosp Psychiatry, 34*(3), 234-241. doi:10.1016/j.genhosppsych.2012.01.007

Bastiaens, L., & Galus, J. (2018). The DSM-5 Self-Rated Level 1 Cross-Cutting Symptom Measure as a Screening Tool. *Psychiatr Q, 89*(1), 111-115. doi:10.1007/s11126-017-9518-7

Grassi-Oliveira R, Cogo-Moreira H, Salum GA, Brietzke E, Viola TW, Manfro GG, Kristensen CH, Arteche AX. Childhood Trauma Questionnaire (CTQ) in Brazilian samples of different age groups: findings from confirmatory factor analysis. PLoS One. 2014 Jan 27;9(1):e87118. doi: 10.1371/journal.pone.0087118. PMID: 24475237; PMCID: PMC3903618.

Johnson, J. G., Harris, E. S., Spitzer, R. L., & Williams, J. B. (2002). The patient health questionnaire for adolescents: validation of an instrument for the assessment of mental disorders among adolescent primary care patients. *J Adolesc Health, 30*(3), 196-204. doi:10.1016/s1054-139x(01)00333-0

Kaufman J, Birmaher B, Brent D, Rao U, Flynn C, Moreci P, Williamson D, Ryan N. Schedule for Affective Disorders and Schizophrenia for School-Age Children-Present and Lifetime Version (K-SADS-PL): initial reliability and validity data. J Am Acad Child Adolesc Psychiatry. 1997 Jul;36(7):980-8. doi: 10.1097/00004583-199707000-00021. PMID: 9204677.

Kieling, C., Buchweitz, C., Caye, A., Manfro, P., Pereira, R., Viduani, A., . . . Mondelli, V. (2021). The Identifying Depression Early in Adolescence Risk Stratified Cohort (IDEA-RiSCo): Rationale, Methods, and Baseline Characteristics. *Front Psychiatry, 12*, 697144. doi:10.3389/fpsyt.2021.697144

Krämer A, Green J, Pollard J Jr, Tugendreich S. Causal analysis approaches in Ingenuity Pathway Analysis. Bioinformatics. 2014 Feb 15;30(4):523-30. doi: 10.1093/bioinformatics/btt703. Epub 2013 Dec 13. PMID: 24336805; PMCID: PMC3928520.

Rocha, T. B., Fisher, H. L., Caye, A., Anselmi, L., Arseneault, L., Barros, F. C., . . . Kieling, C. (2021). Identifying Adolescents at Risk for Depression: A Prediction Score Performance in Cohorts Based in 3 Different Continents. *J Am Acad Child Adolesc Psychiatry, 60*(2), 262-273. doi:10.1016/j.jaac.2019.12.004

Rocha, T. B., Graeff-Martins, A. S., Kieling, C., & Rohde, L. A. (2015). Provision of mental healthcare for children and adolescents: a worldwide view. *Curr Opin Psychiatry, 28*(4), 330-335. doi:10.1097/YCO.0000000000000169
